# Efficacy and safety of neoadjuvant immunotherapy in locally advanced resectable esophageal squamous cell carcinoma: a network meta-analysis and real-world study

**DOI:** 10.3389/fimmu.2026.1764960

**Published:** 2026-02-26

**Authors:** Mengfei Sun, Pengjie Yang, Ling Qi, Ting Yang, Weishi Wang, Yongjun Yu, Jingjing Zhang, Benben Zhu, Yong Li

**Affiliations:** 1 College of Pharmacy, Inner Mongolia Medical University, Hohhot, China; 2 Department of Thoracic Surgery, Peking University Cancer Hospital/Affiliated Cancer Hospital of Inner Mongolia Medical University, Hohhot, China; 3 Department of Oncology, National Cancer Center/National Clinical Research Center for Cancer/Cancer Hospital, Peking Union Medical College, Chinese Academy of Medical Sciences, Beijing, China; 4 Department of Cardiothoracic Surgery, Affiliated Hospital of Southwest Medical University, Luzhou, China; 5 Department of Cardio-Thoracic Surgery, The Second Hospital of Chifeng, Chifeng, China; 6 Department of Pharmacy, Peking University Cancer Hospital/Affiliated Cancer Hospital of Inner Mongolia Medical University, Hohhot, China; 7 Department of Thoracic Surgery, National Cancer Center/National Clinical Research Center for Cancer/Cancer Hospital, Peking Union Medical College, Chinese Academy of Medical Sciences, Beijing, China

**Keywords:** esophageal squamous cell carcinoma, immunotherapy, neoadjuvant therapy, network meta-analysis, real-world study

## Abstract

**Introduction:**

This study aimed to explore the efficacy and safety of neoadjuvant immunotherapy combination regimens in locally advanced resectable esophageal squamous cell carcinoma (ESCC), and evaluate the pros and cons of different regimens and their impacts on survival by integrating network meta-analysis (NMA) and real world studies (RWS). ESCC accounts for approximately 90% of global esophageal cancer cases, with over half occurring in China. Although neoadjuvant chemoradiotherapy improves prognosis, unmet clinical needs persist; the optimal neoadjuvant immunotherapy regimen remains controversial. Current large-scale randomized controlled trials (RCTs) suffer from limited sample sizes and fail to adequately reflect the treatment realities of patients in the Chinese real-world setting.

**Methods:**

Systematic searches of databases including PubMed, Embase, Web of Science, and CNKI identified eligible RCTs and cohort studies for NMA. Concurrently, clinical data of 113 such ESCC patients who received neoadjuvant immunotherapy combination treatment followed by surgery at National Cancer Centre of China (January 2021–December 2023) were retrospectively collected, with logistic and Cox regression analyses used to assess associations between factors (e.g., MPR, radiotherapy) and survival.

**Result:**

NMA results showed Sintilimab plus chemoradiotherapy had the highest pathological complete response (pCR) rate, Camrelizumab plus nab-paclitaxel/platinum performed best in major pathological response (MPR) and radical resection with negative surgical margins (R0 resection) rates, and Sintilimab plus nab-paclitaxel/platinum had the lowest adverse event (AE) incidence. Real-world data revealed a significantly higher MPR rate in the Camrelizumab group than the Tislelizumab group (46.9% vs 12.5%, P = 0.0213). Multivariate analysis indicated MPR and primary tumor T response were independent protective factors for overall survival (OS) and progression-free survival (PFS), while neoadjuvant radiotherapy correlated with poorer OS and PFS.

**Conclusion:**

Neoadjuvant immunotherapy combinations (notably Cam+nab-TP) exhibit favorable efficacy in this ESCC subtype, and MPR serves as a reliable surrogate endpoint for long-term survival. The survival benefit of radiotherapy requires careful assessment, and clinical decisions should balance efficacy and safety, as these findings provide evidence for individualized treatment.

**Systematic Review Registration:**

https://www.crd.york.ac.uk/PROSPERO/view/CRD420251174359, identifier CRD420251174359.

## 1 Introduction

Esophageal cancer is one of the most common malignant tumors of the digestive tract, originating from the esophageal epithelium (1). Globally, it causes over 500,000 cancer-related deaths annually and ranks as the 7th most common cancer worldwide (2). The incidence of esophageal cancer varies significantly across countries and regions, with East Asia having the highest incidence—twice the global average. Esophageal cancer is primarily classified into two histological subtypes: esophageal squamous cell carcinoma (ESCC) and esophageal adenocarcinoma (EAC). EAC is the predominant histological subtype in regions with relatively low esophageal cancer incidence, such as Europe and North America. However, globally, ESCC accounts for approximately 90% of all esophageal cancer cases, and over half of these ESCC cases occur in China (3).

Surgery is the primary treatment for resectable esophageal cancer. However, patients treated with surgery alone have poor prognosis, with a 5-year survival rate rarely exceeding 25%—this highlights the need for multidisciplinary comprehensive therapy (4, 5). In recent years, neoadjuvant therapy, a preoperative treatment modality, has not only improved local tumor control but also increased resectability, thereby conferring overall survival benefits to patients. Two pivotal studies—CROSS and NEOCRTEC 5010—established the role of neoadjuvant chemoradiotherapy in the preoperative management of locally advanced resectable ESCC in Western countries and China. Recently, both studies released long-term follow-up data: the 10-year follow-up of the CROSS study showed a 10-year survival benefit of 13% in patients who received neoadjuvant concurrent chemoradiotherapy; meanwhile, the NEOCRTEC 5010 study demonstrated that neoadjuvant chemoradiotherapy improved the 5-year survival rate from 49.1% in patients treated with surgery alone to 59.9%, representing a significant survival advantage over the surgery alone group. Despite these gains, unmet needs remain. For instance, in the CROSS study, the esophageal cancer-specific mortality rate in the experimental group reached 47%, and recurrence rates were 28.6% in the neoadjuvant chemoradiotherapy group versus 35.4% in the surgery alone group. The primary recurrence pattern was systemic hematogenous distant metastasis, which highlights the need to enhance the intensity of systemic therapy to improve disease control (6, 7).

Immunotherapy exerts its anti-tumor effects by specifically breaking tumor immune evasion, stimulating the body’s immune cells, enabling T lymphocytes to re-recognize tumors and restoring their ability to attack tumor cells (8, 9). Immune checkpoint inhibitors have been proven effective in the treatment of various cancers. Results from the KEYNOTE-181 study demonstrated that Pembrolizumab significantly improved overall survival (OS) in patients with advanced esophageal cancer compared with chemotherapy alone (median OS: 8.2 months vs. 7.1 months; Hazard Ratio(HR) (95%CI): 0.78 (0.63, 0.96); P = 0.0095) (10), ushering in the era of immunotherapy for esophageal cancer. At present, immunotherapy has become a first-line treatment option for advanced and metastatic esophageal cancer (10). In recent years, immunotherapy has also been continuously explored in the neoadjuvant setting for esophageal cancer. A growing number of clinical studies on neoadjuvant immunotherapy combined with chemotherapy or chemoradiotherapy have been carried out and have shown promising therapeutic outcomes. Improvements have been achieved both in local tumor downstaging and R0 resection rate, which significantly reduces surgical difficulty (11, 12).

However, controversy remains regarding the optimal neoadjuvant immunotherapy combination regimen for locally advanced esophageal cancer. There is a paucity of large-sample phase III randomized controlled trials(RCTs), and long-term follow-up data remain underreported. Additionally, RCTs have strict inclusion and exclusion criteria, and their results cannot fully represent applicability to patients with locally advanced esophageal cancer in the Chinese real-world setting—who tend to be older on average and have relatively poorer physical status. Furthermore, previous meta-analyses and retrospective studies have typically only compared the efficacy and safety of neoadjuvant immunotherapy versus conventional chemotherapy for esophageal cancer. Therefore, this study employed a network meta-analysis (NMA) approach to investigate the efficacy and safety of different regimens (including immune checkpoint inhibitors, or ICIs, combined with chemotherapy or chemoradiotherapy) as neoadjuvant treatment strategies for esophageal cancer. It also retrospectively analyzed clinical data from 113 patients with locally advanced esophageal cancer who underwent surgery following neoadjuvant immunotherapy combination treatment at the National Cancer Center/Cancer Hospital, Chinese Academy of Medical Sciences between January 2021 and December 2023. The study aims to provide theoretical evidence for identifying the optimal neoadjuvant immunotherapy combination regimen for locally advanced esophageal cancer.

## 2 Materials and methods

### 2.1 Literature search strategy

A comprehensive literature search was conducted using PubMed, Embase, Web of Science, the Cochrane Library, and China National Knowledge Infrastructure (CNKI). The study search terms comprised: “nivolumab OR pembrolizumab OR camrelizumab OR sintilimab OR toripalimab OR tislelizumab OR atezolizumab OR durvalumab OR avelumab OR ipilimumab OR tremelimumab OR lambrolizumab OR cemiplimab OR programmed cell death-1 (PD-1) or programmed cell death ligand 1 (PD-L1) or cytotoxic T-lymphocyte-associated antigen 4 (CTLA-4) or immunotherapy or immune checkpoint inhibitor’, ‘neoadjuvant or preoperative or perioperative’, ‘esophagus or esophageal or esophageal or esophageal”. Search terms are provided in **Supplementary Table 1**. Reference lists of systematic reviews and included studies were manually searched to identify any additional eligible studies.

### 2.2 Inclusion criteria

Studies meeting the following criteria were included in the meta-analysis: (1) Study design: RCTs; (2) Participants: patients with histologically confirmed resectable ESCC, regardless of region, age, or ethnicity; (3) Intervention: studies comparing neoadjuvant immunotherapy combination regimens; (4) Outcomes: Pathological complete response (pCR), major pathological response (MPR), R0 resection rate, treatment-related adverse events (AEs) of any grade, and AEs of grade 3 or higher.

Studies meeting the following criteria were excluded: (1) Patients who received anti-esophageal cancer therapy prior to neoadjuvant treatment; (2) Studies unable to provide at least one of the aforementioned outcome measures; (3) Studies published as case reports, reviews, or expert opinions; (4) Studies exhibiting significant baseline imbalances between patient groups.

### 2.3 Data extraction and quality assessment

Two investigators (SMF and YPJ) independently conducted literature searches, data extraction, and quality assessments. The following information was extracted from included studies: (1) study characteristics, including first author, publication year, study design, sample size, and details regarding interventions and outcomes; (2) baseline characteristics of enrolled patients, such as gender, age, and clinical TNM staging of tumors; (3) Endpoint data, including pCR rate, MPR rate, R0 resection rate, incidence of AEs of any grade, and incidence of ≥3 grade AEs.

The Cochrane Risk of Bias tool 2.0 was employed to assess the methodological soundness and potential biases of the included RCTs. This tool provides a comprehensive analysis across five distinct domains: potential for bias arising from the randomization phase; bias arising from deviation from the planned intervention; bias arising from incomplete reporting of outcomes; bias affecting the impartial assessment of outcomes; and bias in the disclosure of results.

### 2.4 Patients with locally advanced ESCC in the real world

We retrospectively collected clinical data from 113 patients with locally advanced esophageal cancer who underwent surgery following neoadjuvant immunotherapy at the Cancer Hospital, Chinese Academy of Medical Sciences & China National Cancer Center between January 2021 and December 2023. Inclusion criteria were as follows: (1) Histologically or cytologically confirmed resectable ESCC (cT1b-cT2 N+ or cT3-cT4a, any N—suspected involvement of surrounding organs but not definitively cT4b), with complete imaging studies and deemed resectable by thoracic surgeons. The judgment basis for “suspected involvement of surrounding organs” is as follows: contrast-enhanced CT shows a blurred or unclear space between the tumor and adjacent organs, or PET-CT indicates abnormally increased metabolism of adjacent organs, or EUS reveals that the tumor infiltration depth approaches or reaches the capsule of adjacent organs, which is confirmed by two senior thoracic surgeons combined with imaging reports and clinical evaluations to not meet the definite cT4b stage criteria; (2) Age ≥18 years, both sexes eligible; (3) Normal blood, renal, hepatic, cardiac, and pulmonary function; (4) Received neoadjuvant immunotherapy combination treatment prior to surgery. Exclusion criteria included: (1) Previously received other types of neoadjuvant therapy, including but not limited to chemotherapy alone, radiotherapy alone, targeted therapy, and other immunotherapy regimens (such as immune checkpoint inhibitor monotherapy or combination regimens not included in this study).; (2) Prior neoadjuvant therapy of other types; (3) Patients enrolled in prospective studies; (4) Incomplete data or loss to follow-up.

### 2.5 Pathological findings, outcomes and follow-up

Pathological TNM staging was performed according to the 8th edition of the American Joint Committee on Cancer (AJCC) staging system. Pathological specimens from each patient were assessed by two experienced pathologists (not authors of this paper). The primary outcome was MPR. MPR was defined as pathological residual tumor <10% following tumor regression induced by neoadjuvant therapy. pCR was defined as the absence of residual invasive tumor cells in pathological examination of the primary lesion and mediastinal lymph node specimens. R0 resection was defined as radical resection with negative surgical margins (distal, proximal, and circumferential). Postoperative pathological staging (pTNM) was compared with clinical staging (cTNM) based on pre-neoadjuvant imaging (PET-CT or contrast-enhanced CT). A reduction in either the “T” or “N” stage was defined as a T/N Response. Overall survival (OS) was defined as the time from the start of neoadjuvant therapy to death from any cause. Progression-free survival (PFS) was defined as the time from the start of neoadjuvant therapy to disease progression or death from any cause.

Following surgery, patients underwent assessment every three months for the first two years, every six months for the subsequent three years, and annually thereafter. Survival status, disease progression, and further treatments were recorded at each follow-up visit.

### 2.6 Statistical analysis

Data analysis was conducted using Stata 16.0 software. Multivariate NMA was performed within a frequency statistics framework using the ‘NETWORK’ command in Stata. Given the high to moderate heterogeneity prevalent across the included RCTs, the NMA employed a random-effects model. NMA results are presented in a ranking table. Within this table, a 95% confidence interval (CI) for a dichotomous variable that does not include 1 indicates a statistically significant observed difference. For each outcome, we estimated the relative ranking of different treatment options based on their distribution under the Surface Under Cumulative Ranking (SUCRA) probability. The interpretation of SUCRA values should be in conjunction with specific outcome indicators, and the rankings for efficacy and safety are independent of each other. For efficacy-related indicators such as pCR, MPR, and R0 resection rate, a higher SUCRA value indicates a greater probability of the regimen’s efficacy advantage. In contrast, for safety-related indicators such as the incidence of adverse events, a higher SUCRA value signifies an increased risk of adverse events. These two constitute independent evaluation dimensions with no direct correlation. Standardized Mean Differences (SMD) and Odds Ratios (OR) were employed as effect measures. For continuous outcomes, 95% CI were reported; for dichotomous outcomes, OR with 95% CI were presented.

In the retrospective analysis section, clinical and pathological characteristics were described using counts and percentages. We first performed univariate logistic regression analysis. To avoid omitting potential confounding factors or variables with weak but clinically meaningful associations and reduce the risk of type II errors, variables with P < 0.2 were selected for multivariate logistic regression analysis. Subsequently, Cox proportional hazards regression models (with 95% confidence intervals) assessed associations between factors and all-cause mortality risk. Results were reported for unadjusted, minimally adjusted, and fully adjusted models. P values < 0.05 were considered statistically significant. Kaplan-Meier (KM) curves were further employed to compare cumulative mortality rates. All analyses were performed using R 4.2.2 (http://www.Rproject.org; R Foundation, Vienna, Austria) and Free Statistics software (version 2.2.0; Beijing Free Clinical Medical Technology Co., Ltd., Beijing, China). P values < 0.05 were considered statistically significant.

## 3 Result

### 3.1 Search results and study characteristics

**Figure 1:**
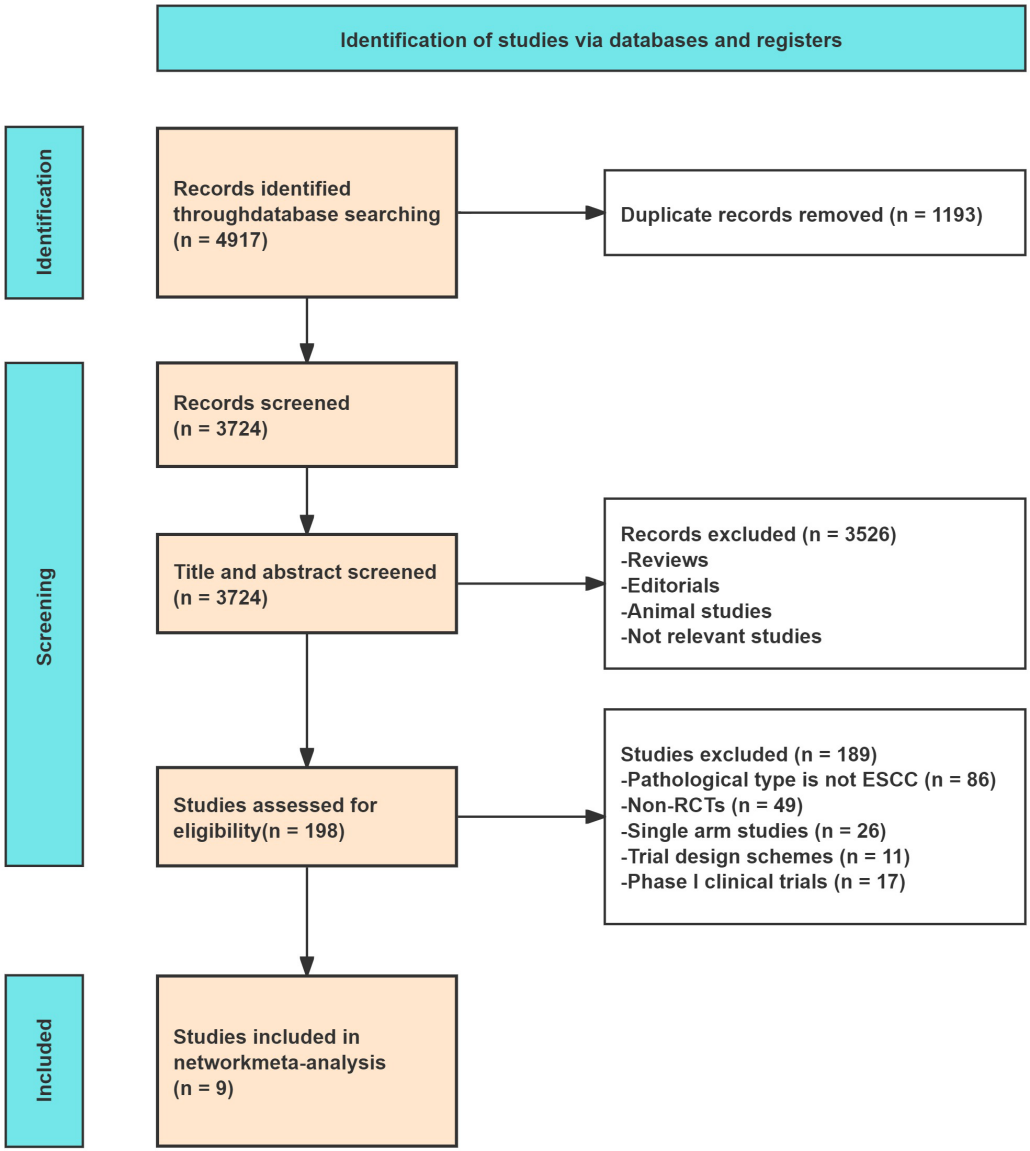
Flow diagram of included studies for this meta-analysis.

During the initial phase of the literature review, comprehensive searches across multiple databases yielded a total of 3724 records. Subsequent abstract screening removed duplicate records and articles irrelevant to our research focus, leading to the selection of 198 studies for detailed full-text assessment. After thorough review, only 9 studies met our strict inclusion criteria (**Figure 1**). The risk of bias assessment graph is provided in **Supplementary Figure 1**. These studies included 10 distinct treatment regimens, categorized as follows: chemotherapy alone (paclitaxel plus platinum (TP), nab-paclitaxel plus platinum (nab-TP)); chemoradiotherapy (nab-paclitaxel plus platinum and radiotherapy (nab-TP+R)); and immunotherapy combination regimens (Camrelizumab+TP, Camrelizumab+nab-TP, Nivolumab+TP, Socazolimab+nab-TP, Toripalimab+TP, Sintilimab+nab-TP, Sintilimab+nab-TP+R). The characteristics of the included studies and study data on outcomes are summarized in **Table 1**, respectively.

**Table 1:** Characteristics of the included studies.

ID	Author	Year	Clinical stage	Group	ICIs	CT	Cycles	Duration of the cycle	Age (years)	Gender males, n (%)	Sample size	Total
1	Renquan Zhang	2023	cT1-4N1-3M0 and cT3-4N0M0	nlCT	Camrelizumab	nab-paclitaxel+cisplatin	NR	NR	65	NR	90	243
				nCT		nab-paclitaxel+cisplatin	NR	NR	65	NR	60	
2	Jianjun Qin	2024	T1b-3N1-3M0 or T3N0M0	nlCT	Camrelizumab	nab-paclitaxel+cisplatin	2	3w	63 (45-75)	116 (87.5)	114	132
				nlCT		paclitaxel+cisplatin	2	3w	63 (44-75)	112 (86.2)	116	130
				nCT		paclitaxel+cisplatin	2	3w	65 (44-75)	104 (80.6)	103	129
3	Jianping Wang	2023	Resectable/Potentially Resectable Locally Advanced ESCC	nlCT	Camrelizumab	Paclitaxel + Carboplatin	4	3w	60.0 ± 3.01	17 (56.7)	30	60
				nCT		Paclitaxel + Carboplatin	2	3w	60.03 ± 2.98	16 (53.3)	30	
4	Heng Jiao	2025	II-III	nlCT	Nivolumab	Paclitaxel + Cisplatin	2	3w	62.59 ± 7.48	50 (83.3)	55	60
				nCT		Paclitaxel + Cisplatin	2	3w	65.10 ± 6.42	26 (86.7)	30	30
5	Yong Li	2023	II–IVa ESCC	nlCT	Socazolimab	nab-paclitaxel + cisplatin	4	3w	61 (53-72)	23 (71.9)	29	32
				nCT		nab-paclitaxel + cisplatin	4	3w	63 (47-74)	28 (87.5)	29	32
6	Fang Liu	2023	IIb–IVa ESCC	nlCT	Toripalimab	Paclitaxel+nedaplatinum	2	3w	56.26 ± 5.11	20 (46.51)	43	86
				nCT		Paclitaxel+nedaplatinum	2	3w	57.98 ± 5.75	18 (41.86)	43	
7	Yan Zheng	2024	T1N1-3M0 - T2-3N0-3M0	nlCT	Toripalimab	Paclitaxel + Cisplatin	2	3w	66 (41-75)	97 (76.38)	102	127
				nCT		Paclitaxel + Cisplatin	2	3w	68 (45-75)	97 (77.6)	88	125
8	w.p.Yuan	2022	cT1b-3N1-3M0,Ct3n0m0	nICT	Camrelizumab	nab-paclitaxel+Cisplatin	4	3w	NR	NR	32	41
				nCRT		nab-paclitaxel+Cisplatin+radiotherapy	2	3w	NR	NR	28	44
9	Xuefeng Leng	2025	cT1N2-3M0 or cT2-4aN0-3M0	nICT	Sintilimab	nab-paclitaxel + Carboplatin	2	NR	NR	89.7	46	46
				nICRT		nab-paclitaxel + Carboplatin+radiotherapy	2	NR	NR		45	45
				nCRT		nab-paclitaxel + Carboplatin+radiotherapy	2	NR	NR		55	55
pCR (%)	MPR (%)	R0 (%)	AEs Incidence (%)	≥3grade AEs Incidence (%)	Neutropenia (%)	Leucopenia (%)	Anemic (%)	Lose hair (%)	Hypokalemia (%)	Hyponatremia (%)	Diarrhea (%)	Rash (%)
27.8	43.3	NR	NR	NR	NR	NR	NR	NR	NR	NR	NR	NR
10.0	26.7	NR	NR	NR	NR	NR	NR	NR	NR	NR	NR	NR
28.0	59.1	99	93.9	10.4	46.2	51.5	31.1	27.2	11.4	10.6	6.1	9.1
15.4	36.2	96	83.1	29.2	36.9	39.2	23.1	23.1	2.3	4.6	6.9	10.8
4.7	20.9	92	83.2	28.8	30.4	32.8	18.4	20	5.6	3.2	4.8	6.4
NR	NR	NR	NR	NR	60	63.3	36.7	30	NR	NR	NR	NR
NR	NR	NR	NR	NR	60	60	40.0	30	NR	NR	NR	NR
15.0	33.3	96	NR	NR	NR	NR	NR	NR	NR	NR	NR	NR
13.3	33.3	97	NR	NR	NR	NR	NR	NR	NR	NR	NR	NR
41.4	69.0	100	100.0	65.6	78.1	78.1	100.0	31.2	34.4	34.4	18.8	1.3
27.6	62.1	97	100.0	62.5	78.1	100	84.4	28.1	6.3	3.1	6.3	0
NR	NR	NR	NR	NR	NR	NR	NR	NR	NR	NR	NR	16.3
NR	NR	NR	NR	NR	NR	NR	NR	NR	NR	NR	NR	20.9
NR	NR	100	100.0	12.5	NR	NR	NR	89.7	15	42.5	48.3	NR
NR	NR	100	100.0	12.4	NR	NR	NR	88.4	13.2	27.1	48.1	NR
40.6	62.5	100	95.1	22.0	14.6	19.5	34.1	NR	NR	NR	NR	NR
35.7	71.4	100	88.6	31.8	29.5	68.2	25.0	NR	NR	NR	NR	NR
13.0	NR	100	50.0	8.7	NR	NR	NR	NR	NR	NR	NR	NR
60.0	NR	100	86.7	31.1	NR	NR	NR	NR	NR	NR	NR	NR
47.3	NR	100	85.5	36.4	NR	NR	NR	NR	NR	NR	NR	NR

nlCT, neoadjuvant chemoimmunotherapy; nCT, neoadjuvant chemotherapy, nICT, neoadjuvant immunochemotherapy, nCRT, neoadjuvant chemoradiotherapy, nICRT, neoadjuvant immunochemoradiotherapy.

### 3.2 Network meta-analysis

#### 3.2.1 Pathological complete response

**Figure 2:**
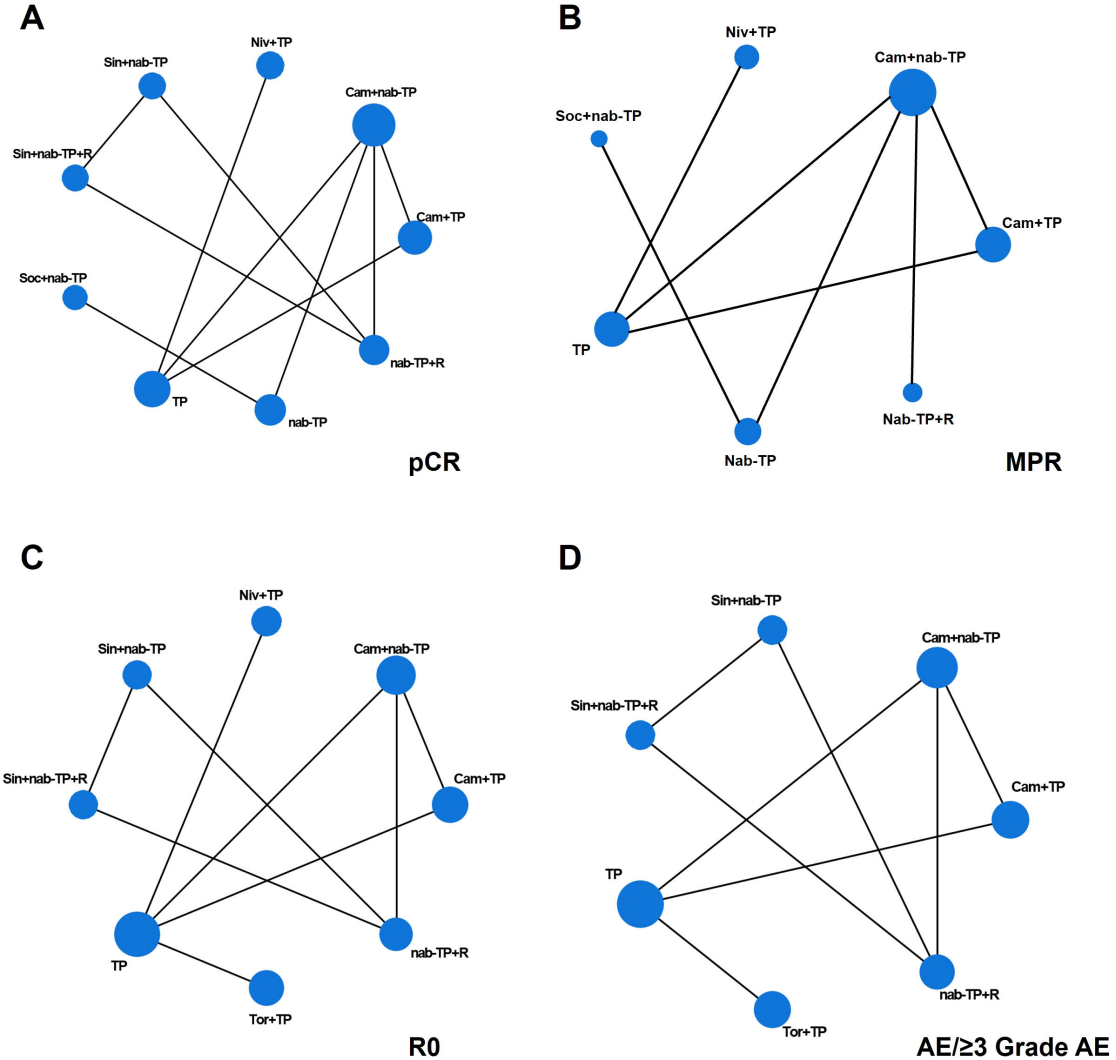
Network diagram of pCR, MPR, R0, AE and ≥3 Grade AE for each treatment regimen in NMA analysis. **(A)** pCR; **(B)** MPR; **(C)** R0; **(D)** AE and ≥ 3 Grade AE. The size of each dot is indicative of the number of studies in which this intervention was used. The lines connecting the dots represent direct comparative studies between two intervention measures.

**Figure 3:**
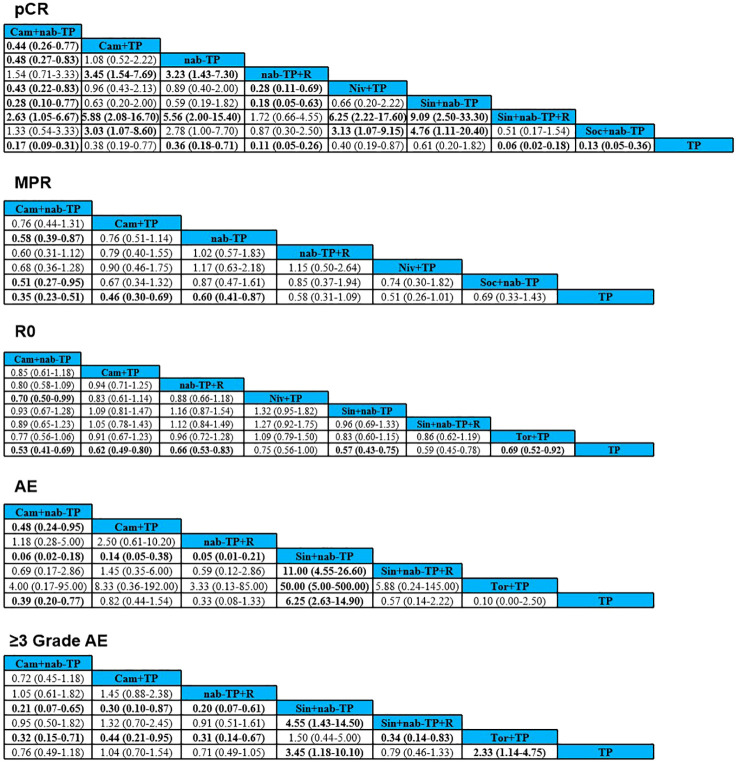
League tables of pCR, MPR, R0, AE and ≥3 Grade AE across treatment regimens in NMA analysis. Bold values in the table indicate statistically significant differences between the two groups (P<0.05); OR, Odds Ratio, which is used to reflect the relative effect strength between treatment regimens. An OR>1 indicates that the former regimen has greater advantages in the corresponding outcome indicators (for efficacy indicators) or higher risks (for safety indicators).

**Figure 4:**
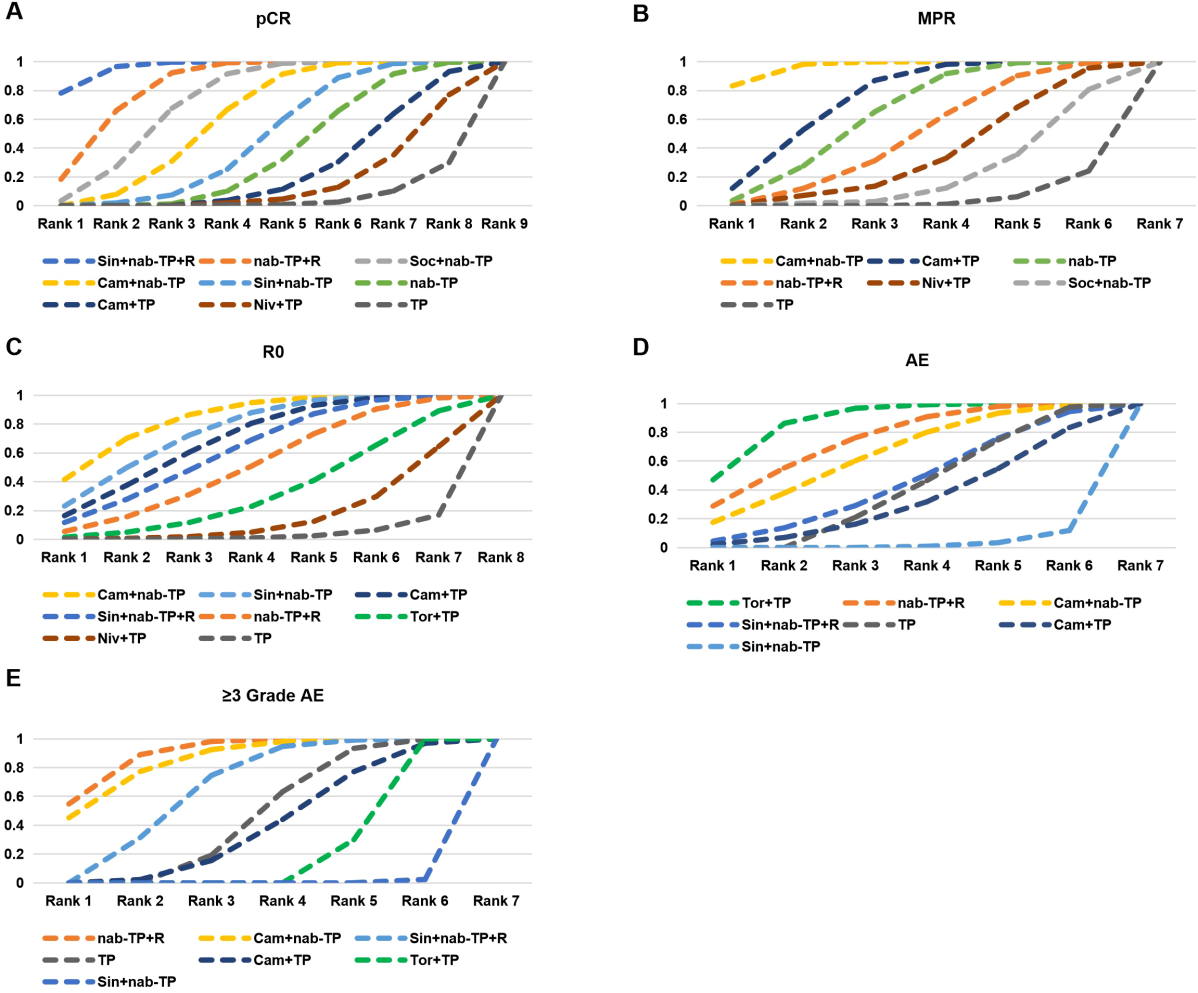
Cumulative ranking probability graphs of pCR, MPR, R0, AE and ≥3 Grade AE across treatment regimens in NMA analysis. **(A)** pCR; **(B)** MPR; **(C)** R0; **(D)** AE; **(E)** ≥ 3 Grade AE. The “ranks” in the figure are calculated based on the Surface Under Cumulative Ranking (SUCRA) values, which are used to quantify the relative advantages and disadvantages of different treatment regimens in corresponding outcome indicators. Specific SUCRA values are provided in the original text. Among them, pCR, MPR, and R0 resection rate are efficacy indicators, with a higher SUCRA value indicating a more significant efficacy advantage, while the incidence of AEs is a safety indicator, where a higher SUCRA value signifies a higher risk of adverse events.

Six studies analyzed the pCR rates of 9 neoadjuvant treatment regimens (**Figure 2A**). The results showed that Sin+nab-TP+R was significantly superior to most regimens, with the following OR and 95% CI: TP (OR = 0.06, 95%CI: 0.02–0.18, P < 0.05), Sin+nab-TP (OR = 9.09, 95%CI: 2.50–33.30, P < 0.05), Niv+TP (OR = 6.25, 95%CI: 2.22–17.60, P < 0.05), nab-TP (OR = 5.56, 95%CI: 2.00–15.40, P < 0.05), Cam+TP (OR = 5.88, 95%CI: 2.08–16.70, P < 0.05), Cam+nab-TP (OR = 2.63, 95%CI: 1.05–6.67, P < 0.05). However, no significant differences were observed between Sin+nab-TP+R and nab-TP+R or Soc+nab-TP. The nab-TP regimen was significantly superior to TP (OR = 0.36, 95%CI: 0.18–0.71, P < 0.05). Additionally, nab-TP+R was significantly superior to both nab-TP and most neoadjuvant chemoimmunotherapy regimens. Cam+nab-TP was significantly superior to Cam+TP, Niv+TP, Sin+nab-TP, nab-TP, and TP, while Soc+nab-TP was significantly superior to Sin+nab-TP, Niv+TP, Cam+TP, and TP (**Figure 3**). Ranked by SUCRA values for pCR rate in descending order: Sin+nab-TP+R (SUCRA = 96.68%), nab-TP+R (SUCRA = 89.75%), Soc+nab-TP (SUCRA = 77.42%), Cam+nab-TP (SUCRA = 62.56%), Sin+nab-TP (SUCRA = 42.97%), nab-TP (SUCRA = 28.56%), Cam+TP (SUCRA = 19.22%), Niv+TP (SUCRA = 12.94%), TP (SUCRA = 5.72%) (**Figure 4A**).

#### 3.2.2 Major pathological response

Five studies analyzed the MPR rates of 7 neoadjuvant treatment regimens (**Figure 2B**). The results showed that Cam+nab-TP was superior to all other regimens, with significant superiority observed over Soc+nab-TP (OR = 0.51, 95%CI: 0.27–0.95, P < 0.05), nab-TP (OR = 0.58, 95%CI: 0.39–0.87, P < 0.05), and TP (OR = 0.35, 95%CI: 0.23–0.51, P < 0.05). Additionally, both Cam+TP and nab-TP were significantly superior to TP (**Figure 3**). Ranked by SUCRA values for MPR rate in descending order: Cam+nab-TP (SUCRA = 96.70%), Cam+TP (SUCRA = 83.33%), nab-TP (SUCRA = 73.33%), nab-TP+R (SUCRA = 61.67%), Niv+TP (SUCRA = 50.00%), Soc+nab-TP (SUCRA = 38.33%), TP (SUCRA = 16.67%) (**Figure 4B**).

#### 3.2.3 R0 resection rate

Six studies analyzed the R0 resection rates of 8 neoadjuvant treatment regimens (**Figure 2C**). The results showed that Cam+nab-TP was significantly superior to Niv+TP (OR = 0.70, 95%CI: 0.50–0.99, P < 0.05) and TP (OR = 0.53, 95%CI: 0.41–0.69, P < 0.05). Furthermore, Cam+TP, nab-TP+R, Sin+nab-TP, and Tor+TP were all significantly superior to TP (**Figure 3**). Ranked by SUCRA values for R0 resection rate in descending order: Cam+nab-TP (SUCRA = 91.38%), Sin+nab-TP (SUCRA = 82.97%), Cam+TP (SUCRA = 76.74%), Sin+nab-TP+R (SUCRA = 70.32%), nab-TP+R (SUCRA = 58.85%), Tor+TP (SUCRA = 41.50%), Niv+TP (SUCRA = 23.89%), TP (SUCRA = 6.35%) (**Figure 4C**).

#### 3.2.4 Safety

Five studies reported the incidence of adverse events (AEs) and adverse events of grade 3 or higher (≥3 grade AEs) associated with neoadjuvant treatment, analyzing a total of 7 neoadjuvant treatment regimens (**Figure 2D**). For overall AEs: Sin+nab-TP was significantly superior to all other regimens; TP was significantly superior to Cam+nab-TP; Cam+TP was significantly superior to Cam+nab-TP; no significant differences were observed between the remaining regimens. For ≥3 grade AEs: Sin+nab-TP still exhibited the best safety profile, with significant superiority over all other regimens except Tor+TP; no significant differences were observed between the remaining regimens (**Figure 3**). Ranked by SUCRA values for AE incidence in descending order: Tor+TP (SUCRA = 95.83%), nab-TP+R (SUCRA = 88.97%), Cam+nab-TP (SUCRA = 83.52%), Sin+nab-TP+R (SUCRA = 56.67%), TP (SUCRA = 51.50%), Cam+TP (SUCRA = 33.08%), Sin+nab-TP (SUCRA = 1.42%). Ranked by SUCRA values for ≥3 grade AE incidence in descending order: nab-TP+R (SUCRA = 95.60%), Cam+nab-TP (SUCRA = 92.30%), Sin+nab-TP+R (SUCRA = 72.30%), TP (SUCRA = 45.80%), Cam+TP (SUCRA = 32.50%), Tor+TP (SUCRA = 2.30%), Sin+nab-TP (SUCRA = 0.20%) (**Figures 4D, E**).

Further analysis revealed that the TP regimen had lower incidences of neutropenia, anemia, leukopenia, and diarrhea but a higher incidence of hypokalemia/hypnatremia. The Tor+TP regimen had higher incidences of hypokalemia, hypnatremia, and alopecia (**Supplementary Figures S2**–**S4**).

### 3.3 Baseline characteristics of real-world patients and logistic regression analysis

This study retrospectively collected clinical data from 125 patients with locally advanced resectable esophageal squamous cell carcinoma. After excluding 12 patients lost to follow-up, 113 patients were finally included for subsequent analysis. All 113 ESCC patients received neoadjuvant immunotherapy combination treatment. The mean age of the patients was 58.3 ± 7.6 years, and more than 50% had comorbidities (such as hypertension and coronary heart disease). Postoperative pathology results showed a pCR rate of 5.3% (6 patients) and an MPR rate of 39.8% (45 patients), both lower than the rates reported in most RCTs (**Table 2**).

**Table 2:** Real-world patient characteristics.

Variables	Number of patient (n = 113)
Sex, n (%)	
Female	14 (12.4)
Male	99 (87.6)
Age, Mean ± SD	58.3 ± 7.6
BMI, Mean ± SD	23.0 ± 3.2
Surgical history, n (%)	
No	75 (66.4)
Yes	38 (33.6)
Smoking, n (%)	
No	35 (31.0)
Yes	78 (69.0)
Drinking, n (%)	
No	31 (27.4)
Yes	82 (72.6)
ECOG PS, n (%)	
0	112 (99.1)
1	1 (0.9)
Nutritional scores, (n)	
1	(85, 75.2%)
2	(19,16.8%)
3	(4, 3.5%)
4	(3, 2.7%)
5	(2, 1.8%)
Tumor location, n (%)	
Lower	43 (38.1)
Middle	32 (28.3)
Middle and Lower	20 (17.7)
Upper	8 (7.1)
Upper and Middle	10 (8.8)
Clinical T stage, n (%)	
T1	6 (5.3)
T2	26 (23.0)
T3	81 (71.7)
Clinical N stage, n (%)	
N0	30 (26.5)
N1	71 (62.8)
N2	12 (10.6)
Clinical M stage, n (%)	
M0	113 (100.0)
Combined diseases, n (%)	
No	56 (49.6)
Yes	57 (50.4)
Combined diseases (Diabetes), n (%)	
No	99 (87.6)
Yes	14 (12.4)
Combined diseases (High blood pressure), n (%)	
No	75 (66.4)
Yes	38 (33.6)
Combined diseases (Coronary heart disease), n (%)	
No	110 (97.3)
Yes	3 (2.7)
Combined diseases (Cerebral infarction), n (%)	
No	108 (95.6)
Yes	5 (4.4)
Combined diseases (Other), n (%)	
No	102 (90.3)
Yes	11 (9.7)
pCR, n (%)	
No	107(94.7)
Yes	6(5.3)
MPR, n (%)	
No	68(60.2)
Yes	45(39.8)

ECOG PS, Eastern Cooperative Oncology Group Performance Status; BMI, Body Mass Index.

Univariate and multivariate Logistic regression analyses were performed to assess the association between various variables and MPR in the 113 patients with locally advanced ESCC. Univariate analysis showed that comorbidities (OR = 0.50, 95%CI: 0.23–1.07, p = 0.073) and the Tis (Tislelizumab) subtype of immune checkpoint inhibitors (OR = 0.21, 95%CI: 0.04–1.09, p = 0.063) were significantly associated with MPR. Variables with P < 0.2 were included in the multivariate analysis, which revealed that comorbidities significantly reduced the MPR rate (OR = 0.41, 95%CI: 0.18–0.94, p = 0.036). Compared with Camrelizumab, Tislelizumab significantly reduced the MPR rate (OR = 0.17, 95%CI: 0.03–0.98, p = 0.047). No other variables showed a significant statistical association in either univariate or multivariate analyses (all p > 0.05) (**Supplementary Table 2**).

**Figure 5:**
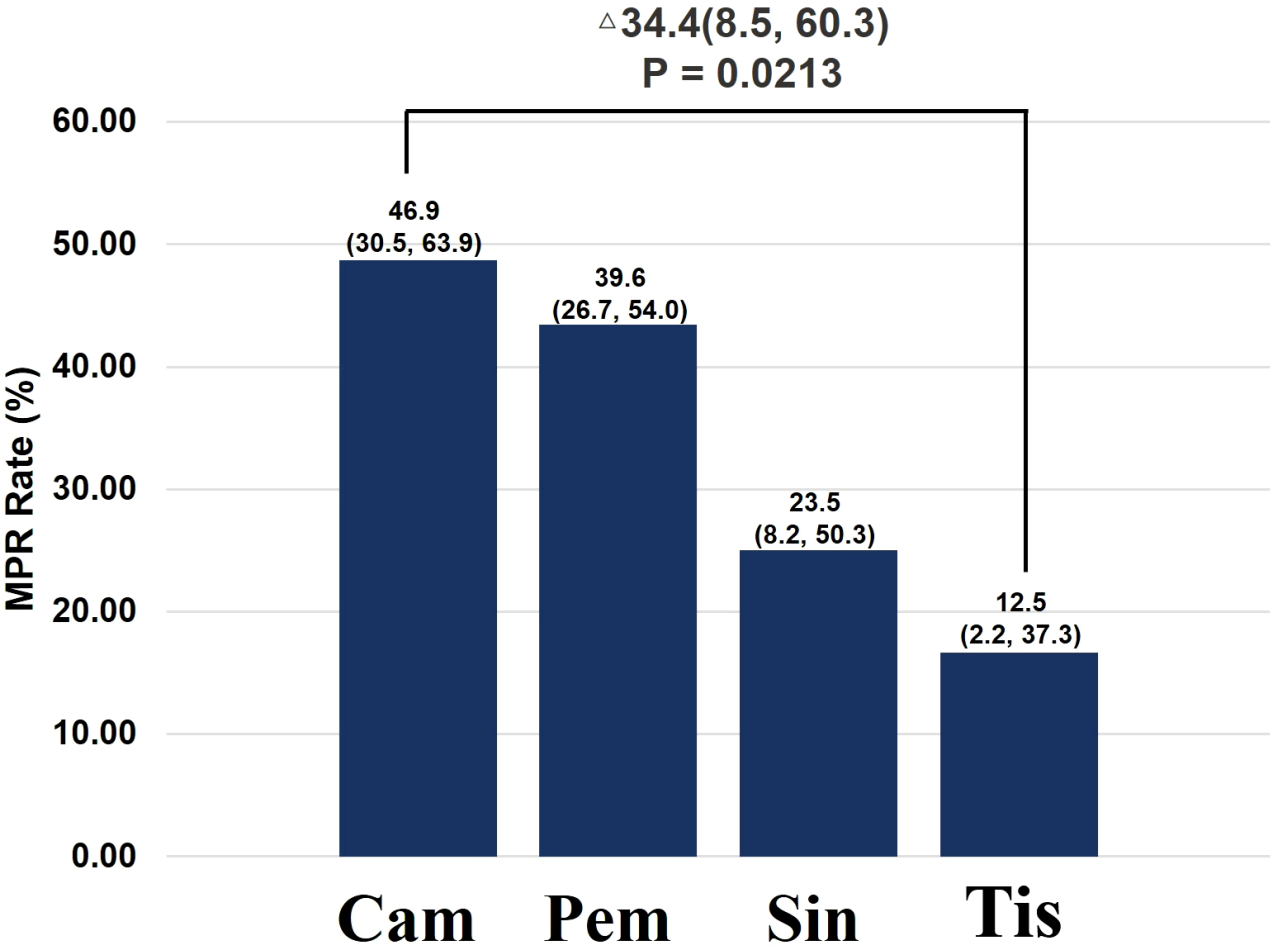
Relationship between ICI type and MPR rate in Real-Word.

Based on the Logistic analysis results, further analysis was conducted on immune checkpoint inhibitors subtypes. The MPR rate in the Cam (Camrelizumab) group was 46.9% (15/32), which was significantly higher than the 12.5% (2/16) in the Tis (Tislelizumab) group (P = 0.0213) (Difference: 34.4%, 95%CI: 8.5–60.3; OR: 6.3, 95%CI: 1.18–33.69). The MPR rates in the Pem (Pembrolizumab) group and Sin (Sintilimab) group were also higher than that in the Tis group, but no statistically significant difference was observed (**Table 3**; **Figure 5**).

**Table 3:** Pathological outcomes of esophageal squamous cell carcinoma patients grouped by ICI type in real-world analysis.

Group	N	MPR, % (95%CI)^a^	Difference (vs Tis group), % (95%CI)^b^	OR(vs Tis group) (95%CI)	P (vs Tis group)^c^
Cam	32	46.9 (30.5, 63.9)	34.4 (8.5, 60.3)	6.3 (1.18, 33.69)	0.0213
Pem	48	39.6 (26.7, 54.0)	27.1 (2.8, 51.4)	4.54 (0.88, 23.44)	0.0611
Sin	17	23.5 (8.2, 50.3)	11 (-18.0, 40.0)	2.13 (0.29, 15.56)	0.4386
Tis	16	12.5(2.2, 37.3)	NA	1 [Reference]	NA

^a^95%CI were calculated based on the Clopper-Pearson method. ^b^95%CI for the stratification factor-adjusted rate differences were derived using the Mantel-Haenszel method. ^c^The Cochran-Mantel-Haenszel test, stratified by clinical stage (I/II versus III), was used to compare between groups. MPR, major pathological response.

### 3.4 Analysis of prognostic factors in real-world patients

**Figure 6:**
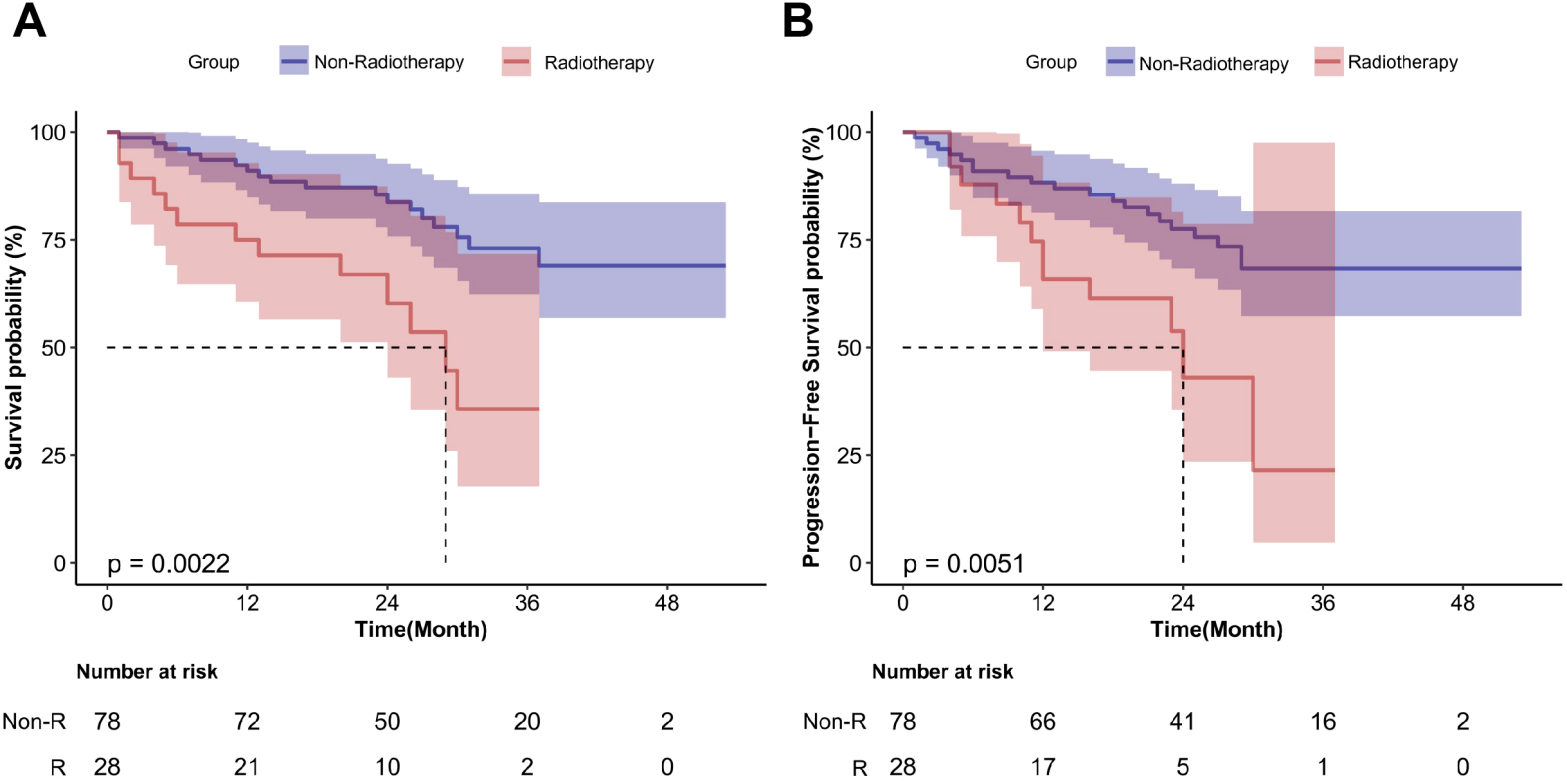
Survival curves for patients with locally advanced esophageal squamous cell carcinoma undergoing neoadjuvant radiotherapy in real-world analysis. **(A)** OS; **(B)** PFS.

**Figure 7:**
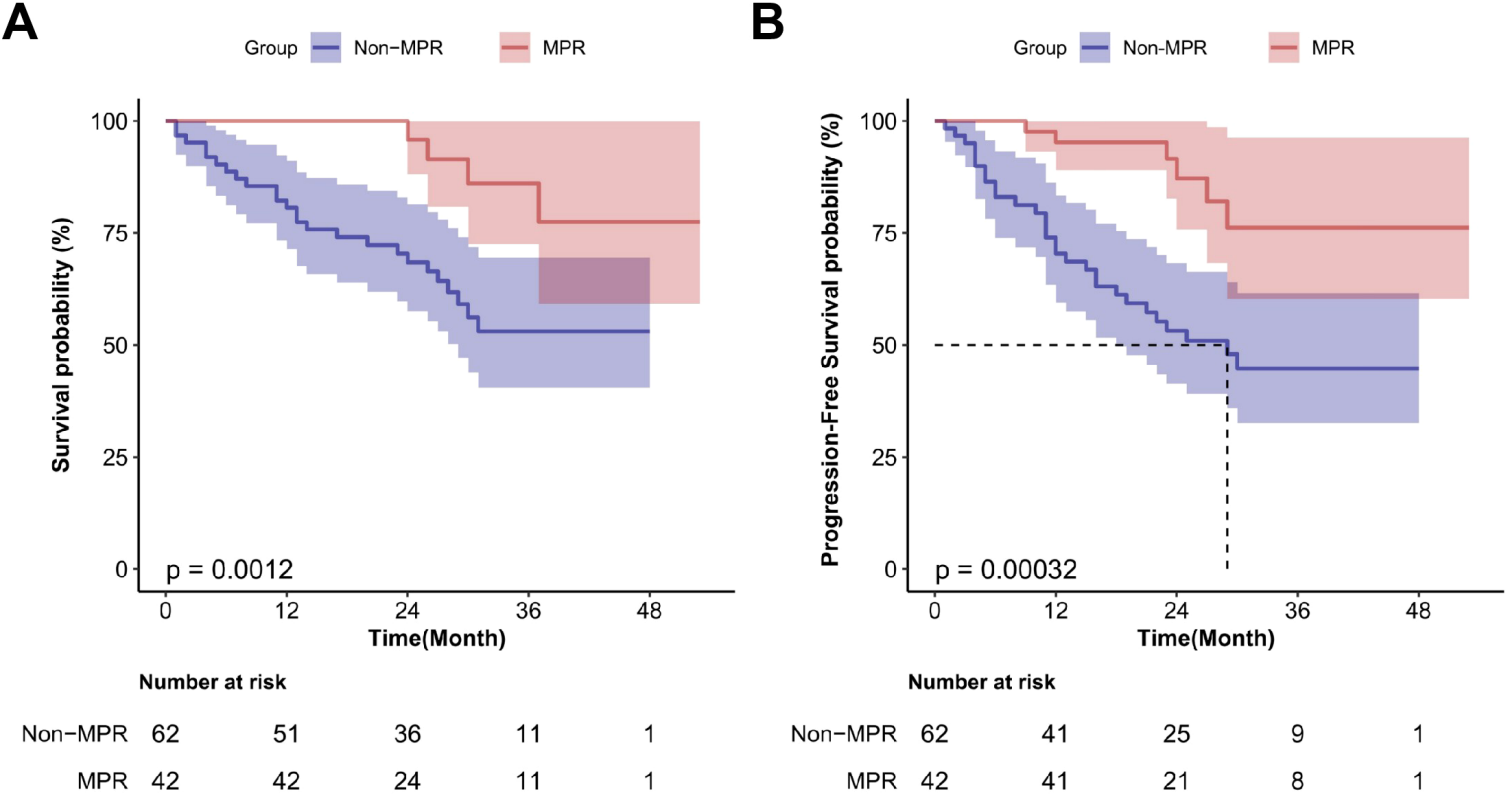
Survival curves for patients with locally advanced esophageal squamous cell carcinoma achieving major pathological response following neoadjuvant therapy in real-world analysis. **(A)** OS; **(B)** PFS.

**Figure 8:**
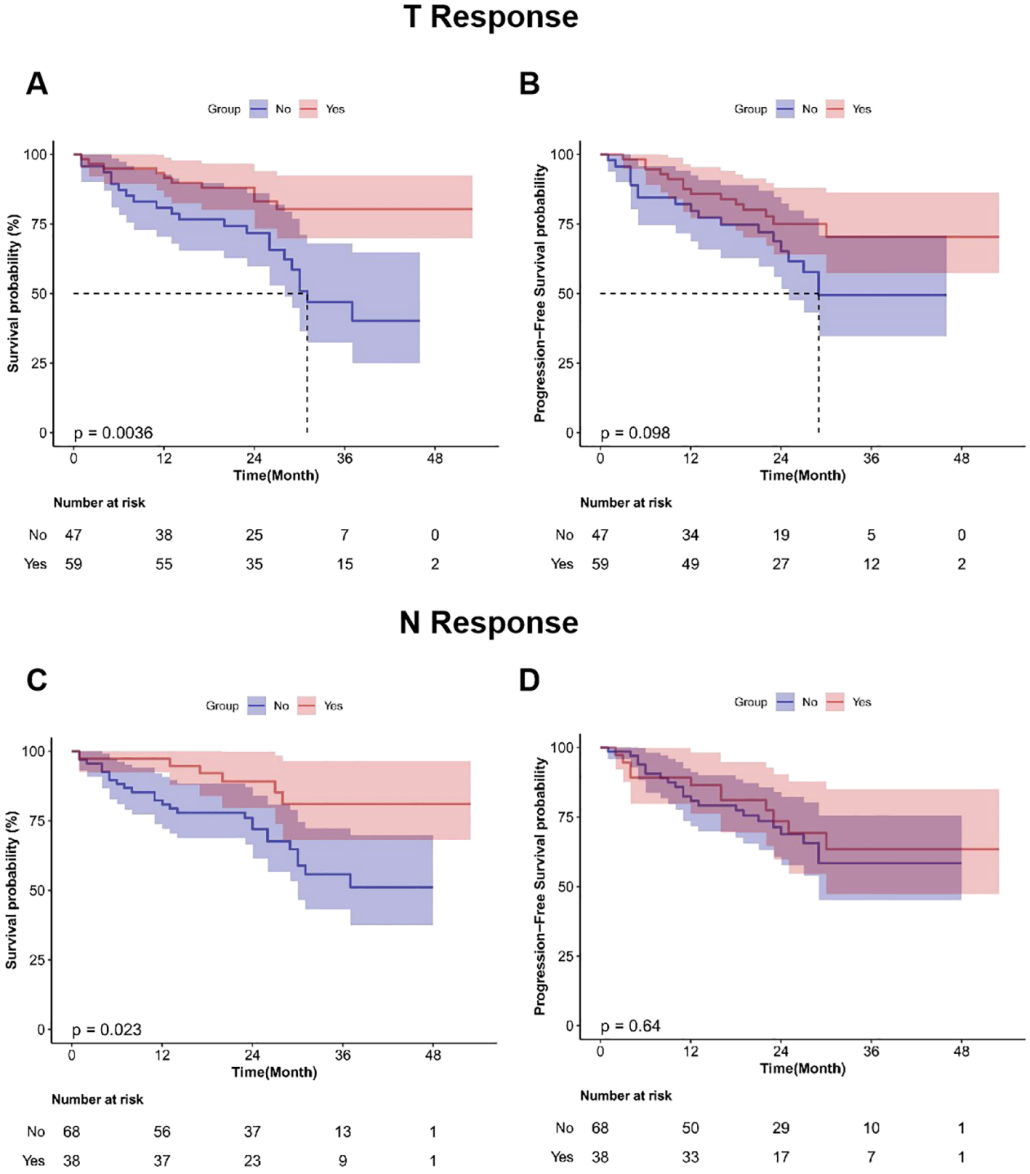
Survival curves for patients with locally advanced esophageal squamous cell carcinoma achieving T/N Response after surgery following neoadjuvant therapy in real-world analysis. **(A)** OS stratified by T Response; **(B)** PFS stratified by T Response; **(C)** OS stratified by N Response; **(D)** PFS stratified by N Response.

RCTs lack long-term survival follow-up data; thus, real-world data were used to further evaluate the associations between various variables and patients’ OS and PFS (**Supplementary Table 3**). The median follow-up period for 113 patients with locally advanced ESCC was 26.0 months. Preoperative neoadjuvant radiotherapy significantly reduced OS (HR = 2.97; 95%CI, 1.44–6.12; P < 0.01) and PFS (HR = 2.75; 95%CI, 1.32–5.71; P < 0.01) (**Figure 6**). Achieving MPR after surgery significantly improved OS (HR = 0.21; 95%CI, 0.07–0.59; P < 0.01) and PFS (HR = 0.23; 95%CI, 0.09–0.55; P < 0.01) (**Figure 7**). Additionally, achieving T Response after neoadjuvant treatment significantly improved OS (HR = 0.34; 95%CI, 0.16–0.73; P < 0.01), and N Response also improved OS (HR = 0.37; 95%CI, 0.15–0.90; P = 0.023). However, neither T nor N Response improved PFS (**Figure 8**).

Multivariate Cox regression analysis was performed to assess the independent effects of MPR, radiotherapy, and T/N Response on survival outcomes (**Tables 4**, **5**). For MPR: after adjusting for clinical stage, clinical T/N stage, radiotherapy, number of lymph nodes dissected, number of treatment cycles, and reconstruction method, MPR remained an independent protective factor for both OS (HR = 0.11; 95%CI, 0.03–0.40; P < 0.01) and PFS (HR = 0.23; 95%CI, 0.09–0.55; P < 0.01). For neoadjuvant radiotherapy: after adjusting for MPR, clinical stage, clinical T/N stage, number of lymph nodes dissected, number of treatment cycles, and reconstruction method, it remained an independent risk factor for both OS (HR = 2.97; 95%CI, 1.44–6.12; P < 0.01) and PFS (HR = 2.91; 95%CI, 1.26–6.72; P = 0.013). For T Response: after adjusting for clinical stage, clinical T/N stage, number of lymph nodes dissected, radiotherapy, number of treatment cycles, and reconstruction method, it was an independent protective factor for both OS (HR = 0.25; 95%CI, 0.10–0.58; P = 0.001) and PFS (HR = 0.42; 95%CI, 0.20–0.86; P = 0.019). In contrast, after adjustment, N Response showed no significant association with either OS or PFS.

**Table 4:** Cox Multivariate Analysis of OS in real-world analysis.

Variable	Model I		Model II		Model III	
	HR (95%CI)	P value	HR (95%CI)	P value	HR (95%CI)	P value
MPR						
No	1 (Ref)		1 (Ref)		1 (Ref)	
Yes	0.21 (0.07,0.59)	0.001	0.22 (0.08~0.64)	0.005	0.11 (0.03~0.4)	**0.001**
Radiotherapy						
No	1 (Ref)		1 (Ref)		1 (Ref)	
Yes	2.97 (1.44~6.12)	0.002	2.92 (1.39~6.1)	0.004	4.91 (1.77~13.63)	**0.002**
T response						
No	1 (Ref)		1 (Ref)		1 (Ref)	
Yes	0.34 (0.16~0.73)	0.004	0.35 (0.16~0.74)	0.006	0.25 (0.1~0.58)	**0.001**
N response						
No	1 (Ref)		1 (Ref)		1 (Ref)	
Yes	0.37 (0.15~0.9)	0.023	0.34 (0.14~0.83)	0.018	0.39 (0.14~1.07)	0.068

Model l, not adjusted; Model II, adjusted for clinical stage; Model III, MPR and T/N Response: adjusted for Clinical N stage, Clinical T stage, Radiotherapy, Number of lymph nodes harvested, Treatment cycles, Reconstruction Pathway and clinical stage. Radiotherapy: adjusted for MPR, Clinical N stage, clinical T stage, number of lymph nodes harvested, treatment cycles, reconstruction pathway and clinical stage. Reconstruction Pathway: adjusted for MPR, Clinical N stage, clinical T stage, radiotherapy, number of lymph nodes harvested, treatment cycles and clinical stage. Bold values indicate statistically significant differences (P < 0.05). HR, Hazard Ratio; CI, Confidence Interval; OS, Overall Survival; MPR, Major Pathological Response.

**Table 5:** Cox Multivariate Analysis of PFS in real-world analysis.

Variable	Model I		Model II		Model III	
	HR (95%CI)	P value	HR (95%CI)	P value	HR (95%CI)	P value
MPR						
No	1 (Ref)		1 (Ref)		1 (Ref)	
Yes	0.23 (0.09,0.55)	<0.001	0.22 (0.09~0.53)	0.001	0.15 (0.06~0.39)	**<0.001**
Radiotherapy						
No	1 (Ref)		1 (Ref)		1 (Ref)	
Yes	2.75 (1.32~5.71)	0.005	2.71 (1.29~5.71)	0.009	2.91 (1.26~6.72)	**0.013**
T response						
No	1 (Ref)		1 (Ref)		1 (Ref)	
Yes	0.56 (0.28~1.12)	0.098	0.52 (0.26~1.05)	0.07	0.42 (0.2~0.86)	**0.019**
N response						
No	1 (Ref)		1 (Ref)		1 (Ref)	
Yes	0.84 (0.4~1.74)	0.638	0.82 (0.39~1.71)	0.594	1.1 (0.5~2.4)	0.815

Model l, not adjusted; Model II, adjusted for clinical stage; Model III, MPR and T/N Response: adjusted for Clinical N stage, Clinical T stage, Radiotherapy, Number of lymph nodes harvested, Treatment cycles, Reconstruction Pathway and clinical stage. Radiotherapy: adjusted for MPR, Clinical N stage, clinical T stage, number of lymph nodes harvested, treatment cycles, reconstruction pathway and clinical stage. Reconstruction Pathway: adjusted for MPR, Clinical N stage, clinical T stage, radiotherapy, number of lymph nodes harvested, treatment cycles and clinical stage. Bold values indicate statistically significant differences (P < 0.05). HR, Hazard Ratio; CI, Confidence Interval; OS, Overall Survival; MPR, Major Pathological Response.

## 4 Discussion

This study pioneeringly employed a combination of NMA and retrospective real-world data to comprehensively evaluate the efficacy and safety of immune checkpoint inhibitors combined with chemotherapy or chemoradiotherapy in the neoadjuvant treatment of locally advanced resectable ESCC. From a macroscopic perspective, NMA provided indirect comparisons and ranking of different treatment strategies; from the clinical practice perspective, real-world data validated the real-world efficacy of these regimens and uncovered their influencing factors. The combination of the two establishes a more three-dimensional and comprehensive evidence base, and also offers valuable reference for clinical individualized decision-making.

First, our NMA results revealed significant heterogeneity in pathological response across different regimens. The Sintilimab+nab-TP+R regimen exhibited the best performance in terms of pCR rate (SUCRA = 96.68%), which aligns with the trend of high pCR rate (55.6%) observed with pembrolizumab plus chemoradiotherapy in Study PALACE-1. This confirms the great potential of immunotherapy combined with chemoradiotherapy in maximizing tumor regression. Radiotherapy can induce the release of more tumor-associated antigens by killing tumor cells; it can also upregulate the expression of PD-L1 and recruit more T lymphocytes, thereby enhancing the efficacy of immunotherapy. Meanwhile, immunotherapy can strengthen radiotherapy-induced immune activation, increase the abscopal effect, and eliminate distant micrometastases (13, 14). However, notably, our real-world data showed that the addition of neoadjuvant radiotherapy was significantly associated with poorer OS and PFS.

A recent large-scale multicenter retrospective study from China also demonstrated that although the MPR rate in the neoadjuvant chemoradiotherapy group was significantly higher than that in the neoadjuvant chemoimmunotherapy group, the neoadjuvant chemoimmunotherapy group achieved better OS and disease-free survival (DFS) than the neoadjuvant chemoradiotherapy group regardless of whether adjuvant immunotherapy was administered. (13.5% vs. 25.0%) (15). This result is not truly contradictory to the finding of the present study that MPR is an independent protective factor for OS/PFS, but rather affected by the confounding effects of multiple clinical and study design factors. First, differences in the baseline characteristics of patients: Notably, however, the chemo-radio-immunotherapy triplet regimen is not the preferred neoadjuvant strategy in real-world clinical practice for ESCC in China. Neoadjuvant radiotherapy is often associated with an increased risk of perioperative complications and greater surgical difficulty (16, 17). Our NMA also showed that the incidences of AEs and ≥3 grade AEs in neoadjuvant immunotherapy plus chemotherapy regimens were generally lower than those in radiotherapy-containing regimens; Sintilimab+nab-TP, in particular, exhibited the most favorable safety profile. Therefore, for patients with locally advanced esophageal cancer, neoadjuvant chemotherapy plus immunotherapy (with better safety) is often prioritized before surgery. Patients with a good treatment response typically opt for direct surgery, while those with more complex conditions, higher tumor burden, or resistance to prior treatment receive sequential neoadjuvant radiotherapy—this contributes to a higher risk of postoperative recurrence and metastasis in the latter group. Second, interference of radiotherapy on treatment tolerance and subsequent therapy: A Dutch study involving 4025 esophageal cancer patients treated with the CROSS regimen showed that severe adverse effects from radiotherapy reduced the chemotherapy completion rate, which in turn led to shortened survival in patients who did not complete chemotherapy (for EAC: 31.8 vs. 42 months, P = 0.0018; for ESCC: 85.6 vs. 57.2 months, P = 0.22) (18). In the ESOPEC study, the chemotherapy completion rate in the CROSS cohort was only 68%, and this cohort was significantly less effective than the FLOT cohort in controlling distant metastases (e.g., liver, lung, and peritoneal metastases), with a significantly higher 3-year distant recurrence rate (31.5% vs. 47.2%) (19). This indicates that chemotherapy, as a systemic treatment, can more effectively eliminate potential micrometastases in the body. Therefore, we conclude that although neoadjuvant radiotherapy can synergistically enhance the efficacy of immunotherapy and chemotherapy, its severe adverse effects impair the completion of treatment regimens, thereby leading to poorer prognosis. In summary, the protective effect of MPR on survival is a general rule, but the survival disadvantage of the radiotherapy group stems from the combined confounding factors of “adverse baseline prognosis + decreased treatment tolerance + insufficient control of distant metastasis,” rather than the failure of MPR’s protective effect. Further analysis of the recurrence patterns in the two groups showed that the neoadjuvant chemoimmunotherapy group had a lower overall recurrence rate (23.7% vs. 35.7%) and a lower distant metastasis rate (13.5% vs. 25.0%) (15), which also confirms the key impact of systemic treatment completion rate on long-term survival. These findings help clinicians strike an optimal balance between efficacy and safety based on patients’ baseline conditions and tolerance.

Among neoadjuvant immunotherapy plus chemotherapy regimens, the Camrelizumab plus nab-paclitaxel and platinum (Camrelizumab+nab-TP) regimen performed best in both MPR rate and R0 resection rate (SUCRA values both exceeding 90%), demonstrating excellent efficacy. This finding was strongly validated by our real-world data: the MPR rate in the Camrelizumab group was as high as 46.9%, significantly higher than that in the Tislelizumab group (12.5%), and comparable to the MPR rate observed with Pembrolizumab. A real-world study pooling data from 987 patients with advanced esophageal cancer treated with Camrelizumab-containing regimens also showed that patients with older age and poorer performance status could still benefit from Camrelizumab treatment (20). Additionally, compared with conventional paclitaxel, nab-paclitaxel has shown advantages across multiple outcomes in terms of efficacy and safety—findings that have been confirmed by multiple meta-analyses and multicenter retrospective studies (21, 22). This superiority may be attributed to nab-paclitaxel’s superior tumor targeting, higher drug permeability, and lower risk of allergic reactions, which provides new insights for clinical medication selection.

MPR and T Response were independent protective factors for patients’ OS and PFS, whereas N Response did not exhibit equivalent prognostic value. This finding holds profound significance for understanding the efficacy assessment of neoadjuvant therapy and the selection of clinical trial endpoints. Multivariate Cox regression analysis demonstrated that achieving MPR was significantly associated with survival benefits. After adjusting for multiple confounding factors (including clinical stage, radiotherapy, and surgical approach), the risk of death in patients who achieved MPR was significantly reduced to 11% of that in non-MPR patients (OS: HR = 0.11, P = 0.001), and the risk of disease progression was reduced to 15% of that in non-MPR patients (PFS: HR = 0.15, P<0.001). To date, multiple meta-analyses and single- and multi-center retrospective studies have shown that MPR is an independent factor associated with improved patient survival and reduced time to disease recurrence (15, 23–25). Therefore, we conclude that MPR can serve as a surrogate endpoint for long-term survival in neoadjuvant immunotherapy for locally advanced esophageal cancer. Additionally, evidence suggests that MPR is a feasible surrogate endpoint for long-term survival; it also provides a more accurate and rapid approach, shortening the time required to evaluate the efficacy of novel chemotherapy and biological therapies in clinical trials (26, 27).

This study selected MPR rather than pCR as the primary study endpoint. A meta-analysis of neoadjuvant immunotherapy for esophageal cancer—including 38 studies—showed that pCR and MPR exhibit consistent results, suggesting that MPR is substitutable for pCR (28). Further analysis of neoadjuvant treatment response patterns revealed that achieving T Response is a strong independent predictor of OS and PFS (OS: HR = 0.25, P = 0.001; PFS: HR = 0.42, P = 0.019), whereas lymph node response or N Response shows no association with prognosis. This indicates that the depth of primary tumor regression may play a more dominant role in prognostic prediction compared with lymph node conversion to negative status. Previous studies also demonstrate that, compared with patients without tumor downstaging, treatment responders with tumor downstaging have significantly improved 5-year survival rates (52.5% vs 12.6%; P<0.001), a higher rate of clear surgical margins at resection (74% vs 40%; P<0.001), a lower isolated local recurrence rate (6% vs 13%; P = 0.030), and a lower systemic metastatic recurrence rate (19% vs 29%; P = 0.027) (29). For N Response, however, even if neoadjuvant therapy eliminates radiologically detectable lymph node metastases, micrometastases or isolated tumor cells may not be completely eradicated. These residual cells become the source of long-term recurrence and metastasis, which diminishes the prognostic value of N Response.

This study also has several limitations. First, the RCTs included in the study’s NMA have limitations in both quantity and quality. On one hand, the limited number of included studies results in insufficient data for direct or indirect comparisons between some interventions. On the other hand, some RCTs have quality flaws such as unclear descriptions of randomization methods and incomplete implementation of blinding—these issues may increase the statistical uncertainty of some comparison results and thus reduce the reliability of the conclusions. Second, this study did not include key clinical variables for stratified analysis, nor did it fully explore the potential impacts of factors such as PD-L1 expression levels and number of treatment cycles on the efficacy and safety of interventions. This could limit the applicability of the study conclusions. Third, our study employed a relatively small sample size, and owing to limitations in data collection and analysis, we did not report the goodness-of-fit test results for the multiple logistic regression model. The absence of this information hinders a comprehensive assessment of the model’s predictive performance and data fit, which may compromise the robustness of the associations between variables and MPR.

Despite these limitations, our study provides a comprehensive summary of randomized controlled trials on immunotherapy for locally advanced resectable esophageal squamous cell carcinoma, revealing important insights and guiding future clinical practice.

## Data Availability

The original contributions presented in the study are included in the article/**Supplementary Material.** Further inquiries can be directed to the corresponding author/s.
