# Supplementary material for: Efficacy and safety of neoadjuvant immunotherapy in locally advanced resectable esophageal squamous cell carcinoma: a network meta-analysis and real-world study
Source: Front Immunol. 2026 Feb 26;17:1764960. doi: 10.3389/fimmu.2026.1764960 (PMC12979121; doi:10.3389/fimmu.2026.1764960)

**Table S1 Search strategy**

| **Databases** | **Search items** |
| --- | --- |
| PubMed | ((((((((((((((((((((((((((immunotherapy[Title/Abstract]) OR ("immune checkpoint inhibitors"[Title/Abstract]) OR ("ICI"[Title/Abstract])) OR ("programmed cell death 1 receptor"[Title/Abstract])) OR ("programmed cell death ligand 1"[Title/Abstract])) OR ("cytotoxic T lymphocyte-associated antigen-4 antigen"[Title/Abstract])) OR ("CTLA-4 antigen"[Title/Abstract])) OR (anti-CTLA-4[Title/Abstract])) OR (CTLA-4[Title/Abstract])) OR (anti-PD-1[Title/Abstract])) OR (anti-PD-L1[Title/Abstract])) OR (PD-1[Title/Abstract])) OR (PD-L1[Title/Abstract])) OR (Avelumab[Title/Abstract])) OR (Atezolizumab[Title/Abstract])) OR (Cemiplimab[Title/Abstract])) OR (Camrelizumab[Title/Abstract])) OR (Durvalumab[Title/Abstract])) OR (Ipilimumab[Title/Abstract])) OR (Lambrolizumab[Title/Abstract])) OR (Nivolumab[Title/Abstract])) OR (Pembrolizumab[Title/Abstract])) OR (Sintilimab[Title/Abstract])) OR (Tremelimumab[Title/Abstract])) OR (Tislelizumab[Title/Abstract])) OR (Toripalimab[Title/Abstract])) ) AND ((((Neoadjuvant[Title/Abstract]) OR (Preoperative[Title/Abstract])) OR (Perioperative[Title/Abstract]))) AND (((((((((((((((((("Esophageal Neoplasms"[MeSH Terms]) OR ("Cancer of Esophagus"[Title/Abstract])) OR ("Cancer of the Esophagus"[Title/Abstract])) OR ("Esophageal Cancer"[Title/Abstract])) OR ("Esophagus Cancer"[Title/Abstract])) OR ("esophageal neoplasm"[Title/Abstract])) OR ("Esophagus Neoplasm"[Title/Abstract])) OR ("carcinoma of esophagus"[Title/Abstract])) OR ("Esophageal squamous cell carcinoma"[Title/Abstract])) OR ("Esophageal squamous carcinoma"[Title/Abstract])) OR ("Esophageal squamous cell cancer"[Title/Abstract])) OR ("Esophageal carcinoma"[Title/Abstract])) OR ("esophageal malignancy"[Title/Abstract])) OR (ESCC[Title/Abstract])) OR (Esophagus[Title/Abstract])) OR (Esophageal[Title/Abstract])) OR (oesophagus[Title/Abstract])) OR (oesophageal[Title/Abstract])) |
| EMBASE | (immunotherapy:ti,ab,kw OR 'immune checkpoint inhibitors':ti,ab,kw OR 'ICI':ti,ab,kw OR 'programmed cell death 1 receptor':ti,ab,kw OR 'programmed cell death ligand 1':ti,ab,kw OR 'cytotoxic t lymphocyte-associated antigen-4 antigen':ti,ab,kw OR 'ctla-4 antigen':ti,ab,kw OR 'anti ctla 4':ti,ab,kw OR 'ctla 4':ti,ab,kw OR 'anti pd 1':ti,ab,kw OR 'anti pd l1':ti,ab,kw OR 'pd 1':ti,ab,kw OR 'pd l1':ti,ab,kw OR atezolizumab:ti,ab,kw OR avelumab:ti,ab,kw OR cemiplimab:ti,ab,kw OR camrelizumab:ti,ab,kw OR durvalumab:ti,ab,kw OR ipilimumab:ti,ab,kw OR lambrolizumab:ti,ab,kw OR nivolumab:ti,ab,kw OR pembrolizumab:ti,ab,kw OR sintilimab:ti,ab,kw OR toripalimab:ti,ab,kw OR tremelimumab:ti,ab,kw OR tislelizumab:ti,ab,kw) AND (neoadjuvant:ti,ab,kw OR preoperative:ti,ab,kw OR perioperative:ti,ab,kw) AND ('esophageal neoplasms':ti,ab,kw OR 'cancer of esophagus':ti,ab,kw OR 'cancer of the esophagus':ti,ab,kw OR 'esophageal cancer':ti,ab,kw OR 'esophagus cancer':ti,ab,kw OR 'esophageal neoplasm':ti,ab,kw OR 'esophagus neoplasm':ti,ab,kw OR 'carcinoma of esophagus':ti,ab,kw OR 'esophageal squamous cell carcinoma':ti,ab,kw OR 'esophageal squamous carcinoma':ti,ab,kw OR 'esophageal squamous cell cancer':ti,ab,kw OR 'esophageal carcinoma':ti,ab,kw OR 'esophageal malignancy':ti,ab,kw OR escc:ti,ab,kw OR esophagus:ti,ab,kw OR esophageal:ti,ab,kw OR oesophagus:ti,ab,kw OR oesophageal:ti,ab,kw) |
| Cochrane Library | ((immunotherapy):ti,ab,kw OR (“immune checkpoint inhibitors”):ti,ab,kw OR (ICI):ti,ab,kw OR (“programmed cell death 1 receptor”):ti,ab,kw OR (“programmed cell death ligand 1”):ti,ab,kw OR (“cytotoxic T lymphocyte-associated antigen-4 antigen”):ti,ab,kw OR (“CTLA-4 antigen”):ti,ab,kw OR (anti-CTLA-4):ti,ab,kw OR (CTLA-4):ti,ab,kw OR (anti-PD-1):ti,ab,kw OR (anti-PD-L1):ti,ab,kw OR (PD-1):ti,ab,kw OR (PD-L1):ti,ab,kw OR (Avelumab):ti,ab,kw OR (Atezolizumab):ti,ab,kw OR (Cemiplimab):ti,ab,kw OR (Camrelizumab):ti,ab,kw OR (Durvalumab):ti,ab,kw OR (Ipilimumab):ti,ab,kw OR (Lambrolizumab):ti,ab,kw OR (Nivolumab):ti,ab,kw OR (Pembrolizumab):ti,ab,kw OR (Sintilimab):ti,ab,kw OR (Toripalimab):ti,ab,kw OR (Tislelizumab):ti,ab,kw OR (Tremelimumab):ti,ab,kw) AND ((Neoadjuvant):ti,ab,kw OR (Preoperative):ti,ab,kw OR (Perioperative):ti,ab,kw) AND (("Esophageal Neoplasms"):ti,ab,kw OR ("Cancer of Esophagus"):ti,ab,kw OR ("Cancer of the Esophagus"):ti,ab,kw OR ("Esophageal Cancer"):ti,ab,kw OR ("Esophagus Cancer"):ti,ab,kw OR (“esophageal neoplasm”):ti,ab,kw OR ("Esophagus Neoplasm"):ti,ab,kw OR ("carcinoma of esophagus"):ti,ab,kw OR ("Esophageal squamous cell carcinoma"):ti,ab,kw OR ("Esophageal squamous carcinoma"):ti,ab,kw OR (“Esophageal squamous cell cancer”):ti,ab,kw OR ("Esophageal carcinoma"):ti,ab,kw OR (“esophageal malignancy”):ti,ab,kw OR (ESCC):ti,ab,kw OR (Esophagus):ti,ab,kw OR (Esophageal):ti,ab,kw OR (oesophagus):ti,ab,kw OR (oesophageal):ti,ab,kw) |
| Web of Science | (((((((((((((((((((((((((((TS=(immunotherapy)) OR TS=(“immune checkpoint inhibitors”) OR TS=(“ICI”)) OR TS=(“programmed cell death 1 receptor”)) OR TS=(“programmed cell death ligand 1”)) OR TS=(“cytotoxic T lymphocyte-associated antigen-4 antigen”)) OR TS=(“CTLA-4 antigen”)) OR TS=(anti-CTLA-4)) OR TS=(CTLA-4)) OR TS=(anti-PD-1)) OR TS=(anti-PD-L1)) OR TS=(PD-1)) OR TS=(PD-L1)) OR TS=(Durvalumab)) OR TS=(Atezolizumab)) OR TS=(Avelumab)) OR TS=(Camrelizumab)) OR TS=(cemiplimab)) OR TS=(Ipilimumab)) OR TS=(Lambrolizumab)) OR TS=(Nivolumab)) OR TS=(Pembrolizumab)) OR TS=(Sintilimab)) OR TS=(Tremelimumab)) OR TS=(Tislelizumab)) OR TS=(Toripalimab)))) AND ((((TS=(Neoadjuvant)) OR TS=(Preoperative)) OR TS=(Perioperative)) AND (((((((((((((((((((TS=("Esophageal Neoplasms")) OR TS=("Cancer of Esophagus")) OR TS=("Cancer of the Esophagus")) OR TS=("Esophageal Cancer")) OR TS=("Esophagus Cancer")) OR TS=(“esophageal neoplasm”)) OR TS=("Esophagus Neoplasm")) OR TS=("carcinoma of esophagus")) OR TS=("Esophageal squamous cell carcinoma")) OR TS=("Esophageal squamous carcinoma")) OR TS=(“Esophageal squamous cell cancer”)) OR TS=("Esophageal carcinoma")) OR TS=(“esophageal malignancy”)) OR TS=(ESCC)) OR TS=(Esophagus)) OR TS=(Esophageal)) OR TS=(oesophagus)) OR TS=(oesophageal)))) |
| CNKI | TKA=(免疫 + 度伐利尤单抗 + 阿替利珠单抗 + 阿维单抗 + 帕博利珠单抗 + 纳武利尤单抗 + 特瑞普利单抗 + 替雷利珠单抗 + 卡瑞丽珠单抗 + 信迪力单抗 + 替西木单抗 + 伊匹单抗 + 派姆单抗 + 西米普利单抗) * (新辅助 + 围术期 + 术前) * (食管癌 + 食管鳞状细胞癌) |
|  |  |

**Table S2 Real-Word Logistic analyze**

|  | **Univariable** | | **Multivariable** | |
| --- | --- | --- | --- | --- |
| **Variable** | **OR (95%CI)** | **P value** | **OR (95%CI)** | **P value** |
| **Sex** |  |  |  |  |
| Female | 1 [Reference] | NA |  |  |
| Male | 0.87 (0.28~2.69) | 0.804 |  |  |
| **Age** |  |  |  |  |
| ＜58 | 1 [Reference] | NA |  |  |
| ≥58 | 1.63 (0.76~3.49) | 0.206 |  |  |
| **BMI** | 1.01 (0.89~1.14) | 0.897 |  |  |
| **Surgical History** |  |  |  |  |
| No | 1 [Reference] | NA | 1 [Reference] | NA |
| Yes | 0.49 (0.21~1.13) | 0.095 | 0.49 (0.2~1.2) | 0.119 |
| **Smoking** |  |  |  |  |
| No | 1 [Reference] | NA |  |  |
| Yes | 1.41 (0.61~3.22) | 0.421 |  |  |
| **Drinking** |  |  |  |  |
| No | 1 [Reference] | NA |  |  |
| Yes | 1.13 (0.46~2.78) | 0.784 |  |  |
| **Tumor Location** |  |  |  |  |
| Lower | 1 [Reference] | NA |  |  |
| Middle | 1.31 (0.52~3.34) | 0.568 |  |  |
| Middle and Lower | 1.12 (0.38~3.34) | 0.832 |  |  |
| Upper | 1.01 (0.21~4.81) | 0.988 |  |  |
| Upper and Middle | 1.12 (0.28~4.6) | 0.87 |  |  |
| **Nutritional Scores** |  |  |  |  |
| 1 | 1 [Reference] | NA |  |  |
| 2 | 0.63 (0.22~1.81) | 0.389 |  |  |
| 3 | 0.45 (0.05~4.54) | 0.501 |  |  |
| 4 | 2.72 (0.24~31.19) | 0.421 |  |  |
| **Clinical stage** |  |  |  |  |
| Ⅰ | 1 [Reference] | NA |  |  |
| Ⅱ | 0.43 (0.07~2.63) | 0.362 |  |  |
| Ⅲ | 0.25 (0.04~1.47) | 0.125 |  |  |
| **Clinical T stage** |  |  |  |  |
| cT1 | 1 [Reference] | NA |  |  |
| cT2 | 0.43 (0.07~2.76) | 0.373 |  |  |
| cT3 | 0.28 (0.05~1.62) | 0.154 |  |  |
| **Clinical N stage** |  |  |  |  |
| cN0 | 1 [Reference] | NA |  |  |
| cN1 | 0.8 (0.34~1.91) | 0.619 |  |  |
| cN2 | 0.93 (0.24~3.62) | 0.921 |  |  |
| **Combined Diseases** |  |  |  |  |
| No | 1 [Reference] | NA | 1 [Reference] | NA |
| Yes | 0.5 (0.23~1.07) | 0.073 | 0.41 (0.18~0.94) | 0.036 |
| **Combined Diseases** |  |  |  |  |
| **Diabetes** |  |  |  |  |
| No | 1 [Reference] | NA |  |  |
| Yes | 0.57 (0.17~1.93) | 0.363 |  |  |
| **High blood pressure** |  |  |  |  |
| No | 1 [Reference] | NA |  |  |
| Yes | 0.59 (0.26~1.34) | 0.205 |  |  |
| **Coronary heart disease** |  |  |  |  |
| No | 1 [Reference] | NA |  |  |
| Yes | 0.75 (0.07~8.52) | 0.817 |  |  |
| **ICI** |  |  |  |  |
| Cam | 1 [Reference] | NA | 1 [Reference] | NA |
| Pem | 0.81 (0.34~1.91) | 0.629 | 0.85 (0.34~2.12) | 0.735 |
| Tir | 0.21 (0.04~1.09) | 0.063 | 0.17 (0.03~0.98) | 0.047 |
| Sin | 0.35 (0.1~1.28) | 0.113 | 0.45 (0.11~1.73) | 0.244 |
| **Radiotherapy** |  |  |  |  |
| No | 1 [Reference] | NA |  |  |
| Yes | 1.01 (0.43~2.37) | 0.982 |  |  |
| **Treatment Cycle** |  |  |  |  |
| 2 | 1 [Reference] | NA |  |  |
| 3 | 0.84 (0.33~2.19) | 0.726 |  |  |
| 4 | 0.61 (0.25~1.54) | 0.297 |  |  |
| 5 | 0.41 (0.04~4.21) | 0.453 |  |  |

**Table S3 Cox univariate analysis**

|  | **OS** | | **PFS** | |
| --- | --- | --- | --- | --- |
| **Variable** | **HR(95%CI)** | **P value** | **HR(95%CI)** | **P** **value** |
| **Sex** |  |  |  |  |
| Male | 1 [Reference] |  | 1 [Reference] |  |
| Female | 2.51 (0.6,10.53) | 0.209 | 2.88 (0.69,12.08) | 0.148 |
| **Age** |  |  |  |  |
| ＜57 | 1 [Reference] |  | 1 [Reference] |  |
| ≥57 | 0.64 (0.31,1.32) | 0.225 | 0.87 (0.43,1.74) | 0.693 |
| **BMI** | 0.94 (0.84,1.06) | 0.314 | 0.98 (0.88,1.09) | 0.73 |
| **ECOG PS** |  |  |  |  |
| 1 | 1 [Reference] |  | 1 [Reference] |  |
| 0 | 15.56 (1.91,126.94) | **0.01** | 0.45 (0.14,1.51) | 0.198 |
| **Nutritional Scores** |  |  |  |  |
| 1 | 1 [Reference] |  | 1 [Reference] |  |
| 2 | 1.62 (0.69,3.81) | 0.273 | 0.92 (0.35,2.39) | 0.857 |
| 3 | 0.78 (0.1,5.88) | 0.813 | 0 (0,Inf) | 0.998 |
| 4 | 1.11 (0.15,8.36) | 0.918 | 0 (0,Inf) | 0.998 |
| 5 | 2.12 (0.28,15.84) | 0.463 | 3.44 (0.81,14.67) | 0.094 |
| **Tumor Location** |  |  |  |  |
| Lower | 1 [Reference] |  | 1 [Reference] |  |
| Middle | 0.61 (0.24,1.57) | 0.309 | 0.64 (0.25,1.64) | 0.35 |
| Middle and Lower | 0.92 (0.34,2.46) | 0.868 | 0.95 (0.36,2.55) | 0.925 |
| Upper | 1.17 (0.33,4.16) | 0.807 | 1.08 (0.3,3.85) | 0.903 |
| Upper and Middle | 0.88 (0.25,3.12) | 0.839 | 1.27 (0.41,3.95) | 0.677 |
| **Clinical Stage** |  |  |  |  |
| I | 1 [Reference] |  | 1 [Reference] |  |
| II | 0.62 (0.13,2.91) | 0.543 | (0,Inf) | 0.997 |
| III | 1.11 (0.26,4.75) | 0.886 | (0,Inf) | 0.997 |
| **Clinical T stage** |  |  |  |  |
| T1 | 1 [Reference] |  | 1 [Reference] |  |
| T2 | 1.03 (0.22,4.88) | 0.965 | (0,Inf) | 0.997 |
| T3 | 0.87 (0.2,3.72) | 0.852 | (0,Inf) | 0.997 |
| **Clinical N stage** |  |  |  |  |
| N0 | 1 [Reference] |  | 1 [Reference] |  |
| N1 | 1.74 (0.71,4.28) | 0.226 | 0.75 (0.35,1.64) | 0.476 |
| N2 | 0.74 (0.15,3.68) | 0.714 | 0.84 (0.26,2.67) | 0.764 |
| **ICI Type** |  |  |  |  |
| Cam | 1 [Reference] |  | 1 [Reference] |  |
| Pam | 0.78 (0.32,1.88) | 0.575 | 0.61 (0.27,1.35) | 0.22 |
| Sin | 2.31 (0.8,6.68) | 0.123 | 1.49 (0.49,4.51) | 0.476 |
| Tir | 1.33 (0.49,3.62) | 0.57 | 0.45 (0.13,1.57) | 0.213 |
| **Radiotherapy** |  |  |  |  |
| No | 1 [Reference] |  | 1 [Reference] |  |
| Yes | 2.97 (1.44,6.12) | **0.002** | 2.75 (1.32,5.71) | **0.005** |
| **Treatment Cycle** |  |  |  |  |
| 2 | 1 [Reference] |  | 1 [Reference] |  |
| 3 | 0.87 (0.37,2.09) | 0.764 | 1.83 (0.78,4.31) | 0.168 |
| 4 | 0.73 (0.3,1.73) | 0.469 | 1.42 (0.6,3.36) | 0.421 |
| 5 | 0.72 (0.09,5.49) | 0.749 | 0 (0,Inf) | 0.997 |
| **MPR** |  |  |  |  |
| No | 1 [Reference] |  | 1 [Reference] |  |
| Yes | 0.21 (0.07,0.59) | **0.001** | 0.23 (0.09,0.55) | **＜0.001** |
| **T Response** |  |  |  |  |
| No | 1 [Reference] |  | 1 [Reference] |  |
| Yes | 0.34 (0.16,0.73) | **0.004** | 0.56 (0.28,1.12) | 0.098 |
| **N Response** |  |  |  |  |
| No | 1 [Reference] |  | 1 [Reference] |  |
| Yes | 0.37 (0.15,0.9) | **0.023** | 0.84 (0.4,1.74) | 0.638 |
| **Reconstruction Pathway** |  |  |  |  |
| PM | 1 [Reference] |  | 1 [Reference] |  |
| RS | 0.37 (0.11,1.22) | 0.103 | 0.92 (0.39,2.15) | 0.851 |
| **Resection Type** |  |  |  |  |
| Non-R0 | 1 [Reference] |  | 1 [Reference] |  |
| R0 | 0.22 (0.08,0.59) | **0.003** | 0.45 (0.14,1.51) | 0.198 |
| **Number of lymph nodes harvested** | 1.01 (0.99,1.04) | 0.317 | 0.9974 (0.9675,1.0281) | 0.864 |
| **Surgical History** |  |  |  |  |
| No | 1 [Reference] |  | 1 [Reference] |  |
| Yes | 1.77 (0.87,3.61) | 0.117 | 0.88 (0.41,1.91) | 0.753 |
| **Smoking History** |  |  |  |  |
| No | 1 [Reference] |  | 1 [Reference] |  |
| Yes | 1.15 (0.53,2.51) | 0.719 | 1.18 (0.55,2.55) | 0.674 |
| **Drinking History** |  |  |  |  |
| No | 1 [Reference] |  | 1 [Reference] |  |
| Yes | 1.47 (0.63,3.41) | 0.372 | 1.91 (0.79,4.64) | 0.154 |
| **Combined Diseases** |  |  |  |  |
| No | 1 [Reference] |  | 1 [Reference] |  |
| Yes | 1.8 (0.85,3.82) | 0.128 | 0.66 (0.33,1.33) | 0.244 |
| **Diabetes** |  |  |  |  |
| No | 1 [Reference] |  | 1 [Reference] |  |
| Yes | 1.12 (0.43,2.93) | 0.814 | 0.85 (0.3,2.44) | 0.764 |
| **High Blood Pressure** |  |  |  |  |
| No | 1 [Reference] |  | 1 [Reference] |  |
| Yes | 2.02 (1,4.09) | 0.05 | 0.83 (0.39,1.75) | 0.626 |
| **Coronary Heart Disease** |  |  |  |  |
| No | 1 [Reference] |  | 1 [Reference] |  |
| Yes | 2.57 (0.61,10.81) | 0.199 | 3.27 (0.77,13.84) | 0.107 |
| **Cerebral Infarction** |  |  |  |  |
| No | 1 [Reference] |  | 1 [Reference] |  |
| Yes | 1.66 (0.39,6.96) | 0.49 | 0 (0,Inf) | 0.997 |

**Figure S1 Literature Quality Assessment**


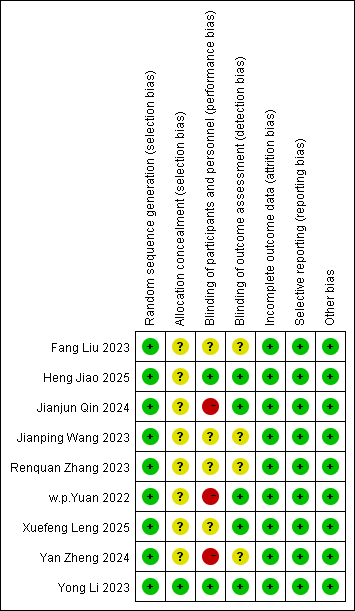


**Figure S2 Network graph for other AEs**


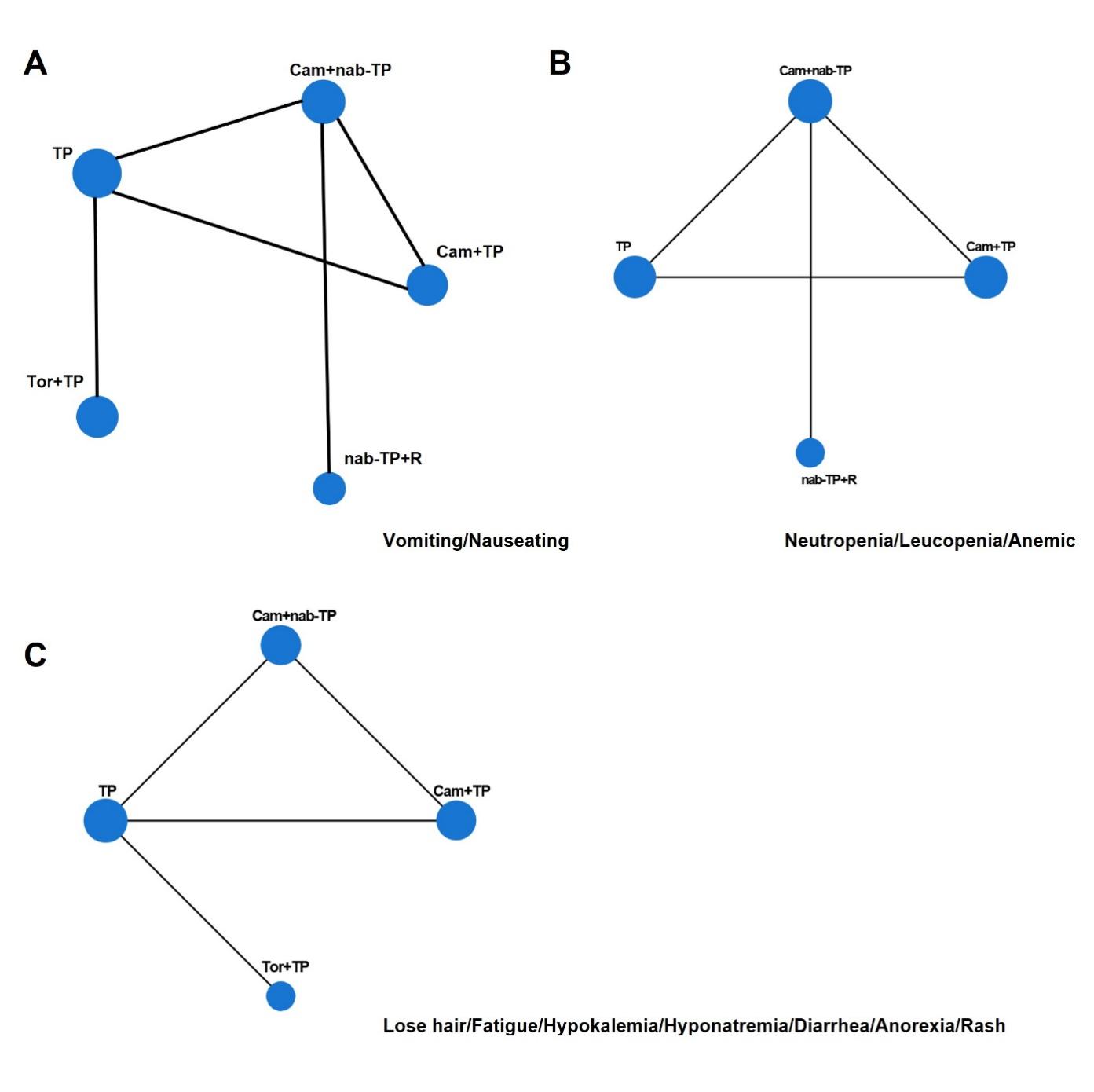


**Figure S3 League Chart other AEs**

**
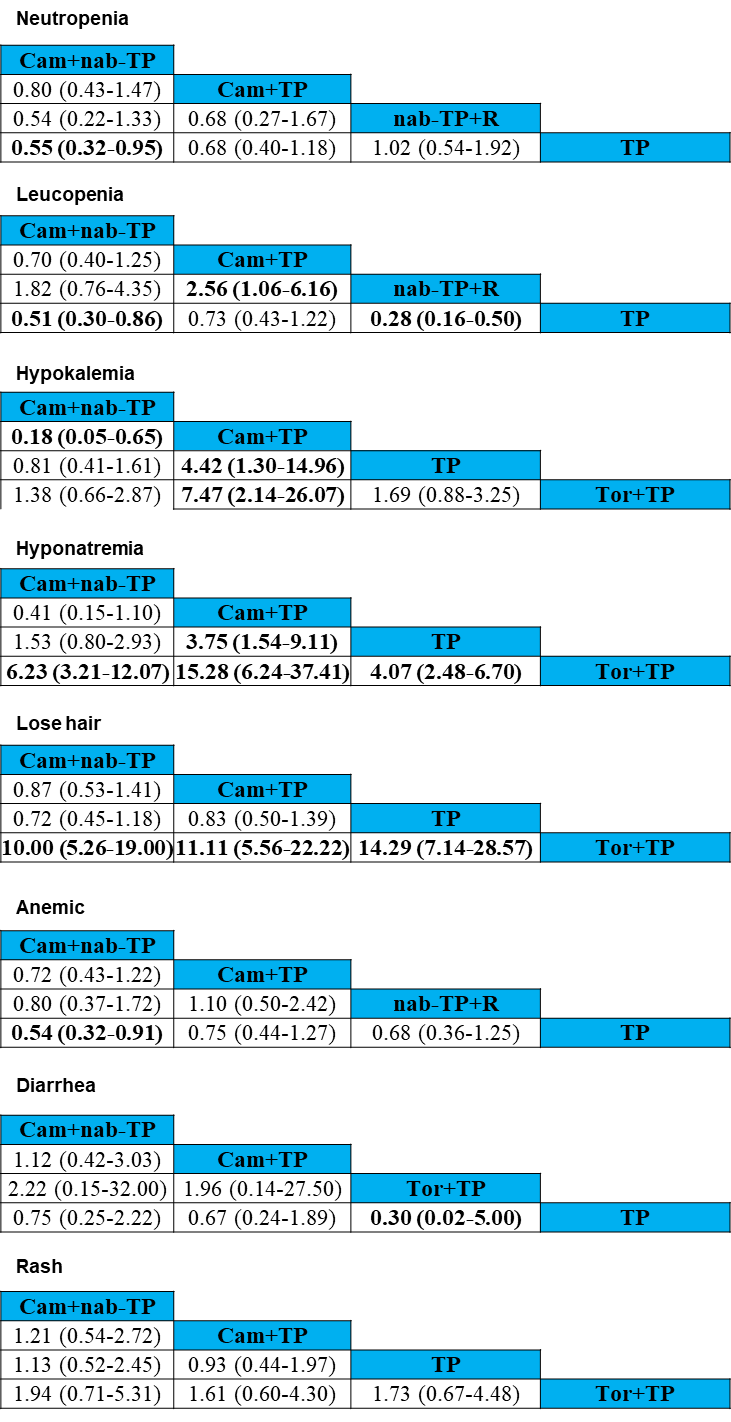
**

**Figure S4 Cumulative ranking probability graphs for other AEs**


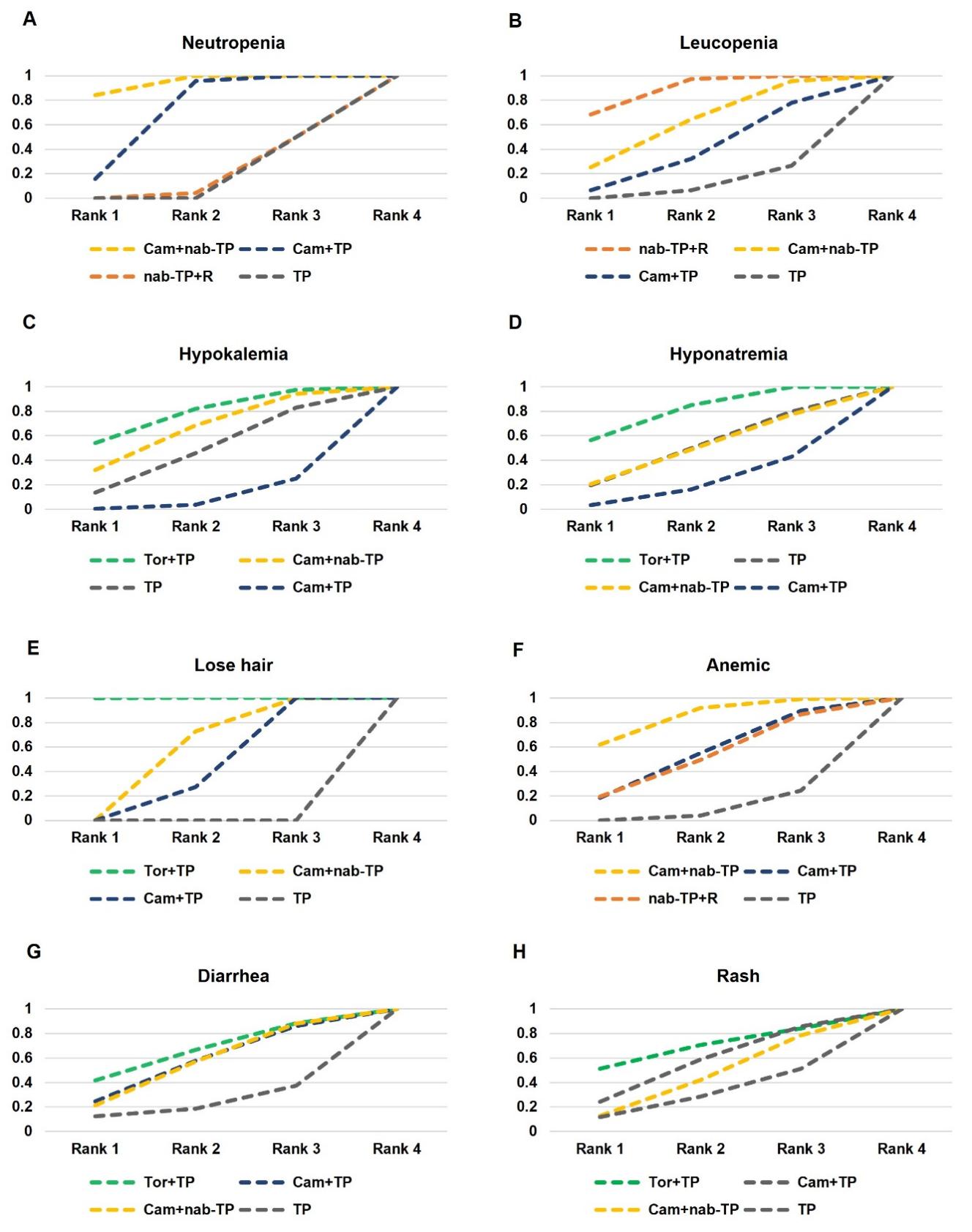

Supplement: Supplementary file 2 [file Table2.docx]
